# Population Analysis of Vibrio cholerae in Aquatic Reservoirs Reveals a Novel Sister Species (*Vibrio paracholerae* sp. nov.) with a History of Association with Humans

**DOI:** 10.1128/AEM.00422-21

**Published:** 2021-08-11

**Authors:** Mohammad Tarequl Islam, Tania Nasreen, Paul C. Kirchberger, Kevin Y. H. Liang, Fabini D. Orata, Fatema-Tuz Johura, Nora A. S. Hussain, Monica S. Im, Cheryl L. Tarr, Munirul Alam, Yann F. Boucher

**Affiliations:** a Department of Biological Sciences, University of Alberta, Edmonton, Alberta, Canada; b Department of Integrative Biology, University of Texas at Austin, Austin, Texas, USA; c Infectious Diseases Division, International Centre for Diarrheal Disease Research, Bangladesh (icddr,b), Dhaka, Bangladesh; d National Center for Emerging and Zoonotic Infectious Diseases, Centers for Disease Control and Prevention, Atlanta, Georgia, USA; e Saw Swee Hock School of Public Health, National University of Singapore, Singapore; Norwegian University of Life Sciences

**Keywords:** *Vibrio cholerae*, cholera, novel species, paracholera, population genomics

## Abstract

Most efforts to understand the biology of Vibrio cholerae have focused on a single group, the pandemic-generating lineage harboring the strains responsible for all known cholera pandemics. Consequently, little is known about the diversity of this species in its native aquatic environment. To understand the differences in the V. cholerae populations inhabiting regions with a history of cholera cases and those lacking such a history, a comparative analysis of population composition was performed. Little overlap was found in lineage compositions between those in Dhaka, Bangladesh (where cholera is endemic), located in the Ganges Delta, and those in Falmouth, MA (no known history of cholera), a small coastal town on the United States east coast. The most striking difference was the presence of a group of related lineages at high abundance in Dhaka, which was completely absent from Falmouth. Phylogenomic analysis revealed that these lineages form a cluster at the base of the phylogeny for the V. cholerae species and were sufficiently differentiated genetically and phenotypically to form a novel species. A retrospective search revealed that strains from this species have been anecdotally found from around the world and were isolated as early as 1916 from a British soldier in Egypt suffering from choleraic diarrhea. In 1935, Gardner and Venkatraman unofficially referred to a member of this group as Vibrio paracholerae. In recognition of this earlier designation, we propose the name *Vibrio paracholerae* sp. nov. for this bacterium. Genomic analysis suggests a link with human populations for this novel species and substantial interaction with its better-known sister species.

**IMPORTANCE** Cholera continues to remain a major public health threat around the globe. Understanding the ecology, evolution, and environmental adaptation of the causative agent (Vibrio cholerae) and tracking the emergence of novel lineages with pathogenic potential are essential to combat the problem. In this study, we investigated the population dynamics of Vibrio cholerae in an inland locality, which is known as endemic for cholera, and compared them with those of a cholera-free coastal location. We found the consistent presence of the pandemic-generating lineage of V. cholerae in Dhaka, where cholera is endemic, and an exclusive presence of a lineage phylogenetically distinct from other V. cholerae lineages. Our study suggests that this lineage represents a novel species that has pathogenic potential and a human link to its environmental abundance. The possible association with human populations and coexistence and interaction with toxigenic V. cholerae in the natural environment make this potential human pathogen an important subject for future studies.

## INTRODUCTION

Vibrio cholerae is the causative agent of cholera, a disease that has shaken human civilization in the past few centuries and continues to be a public health threat, especially to the developing world (1, 2). Its pathogenesis and epidemiology have been extensively studied, but the aquatic part of its life cycle is still not fully understood. Strikingly, few close relatives have been found for this species in recent years, most being initially classified as V. cholerae-like bacteria. One of them was the occasional human pathogen Vibrio mimicus, which was proposed as a new species in 1981 based on phenotypic characteristics (3). Later, genome-based studies established the molecular basis of its importance as a pathogen and close association and exchange of important virulence genes with V. cholerae (4, 5). Two other closely related novel species, *Vibrio* sp. (unofficially named Vibrio parilis) and Vibrio metoecus, were more recently isolated alongside V. cholerae from coastal waters (6, 7) and were found to exchange genetic material with their well-known sister species in aquatic environments (6, 8). Biological information on the close relatives of a dangerous environmental pathogen like V. cholerae is of significance because of their potential as emerging pathogens themselves and their interaction with V. cholerae in their natural habitats. Even though this diverse species is ubiquitous in tropical and temperate coastal waters worldwide, cholera is only caused by a specific lineage of V. cholerae in which the O1 antigen is ancestral, the pandemic-generating (PG) V. cholerae lineage. It is not clear whether aquatic V. cholerae maintains a significantly different population structure in areas where cholera is endemic and not endemic and if this structure is influenced by cooccurring species. This is a crucial gap in our understanding of the factors defining cholera endemicity and driving local and global biogeographic dispersal patterns of V. cholerae. It has recently become possible to investigate the details of the population structure of V. cholerae and its close relatives using a molecular marker based on the single-copy housekeeping gene vibriobactin utilization protein subunit B (*viuB*), which provides subspecies-level resolution (9). This method was used to study a cholera-free region on the east coast of the United States, the Oyster Pond ecosystem (Falmouth, MA), where differences in abundance of individual alleles in particular locations/habitats indicated potential adaptation to ecological conditions at the subspecies level (9, 10). A similar study was performed in V. cholerae populations in an inland location (Dhaka) in Bangladesh where cholera is endemic (9, 11).

Here, to understand the role played by subspecies population structure in disease, we compared the V. cholerae population from inland Bangladesh with that from the east coast of the United States. This revealed that distribution and abundance of major lineages of V. cholerae differ significantly in the two distinct ecosystems. Both globally distributed as well as locally adapted lineages of V. cholerae are found in the two environments studied. One of the most striking differences was the presence of several related lineages in Dhaka that form a divergent clade at the base of the V. cholerae species in a phylogenomic analysis and were completely absent in the coastal United States location. Genomic characterization of these lineages revealed that they form a novel species closely related to, but distinct from, V. cholerae. A revision of recent and decades-old historical isolates related to this novel species indicated that it has been found in similar environments to pandemic V. cholerae for decades and is associated with human infections ranging from septicemia to choleraic diarrhea.

## RESULTS AND DISCUSSION

### Pandemic-related strains increase total V. cholerae abundance in Dhaka and reduce local diversity.

**FIG 1:**
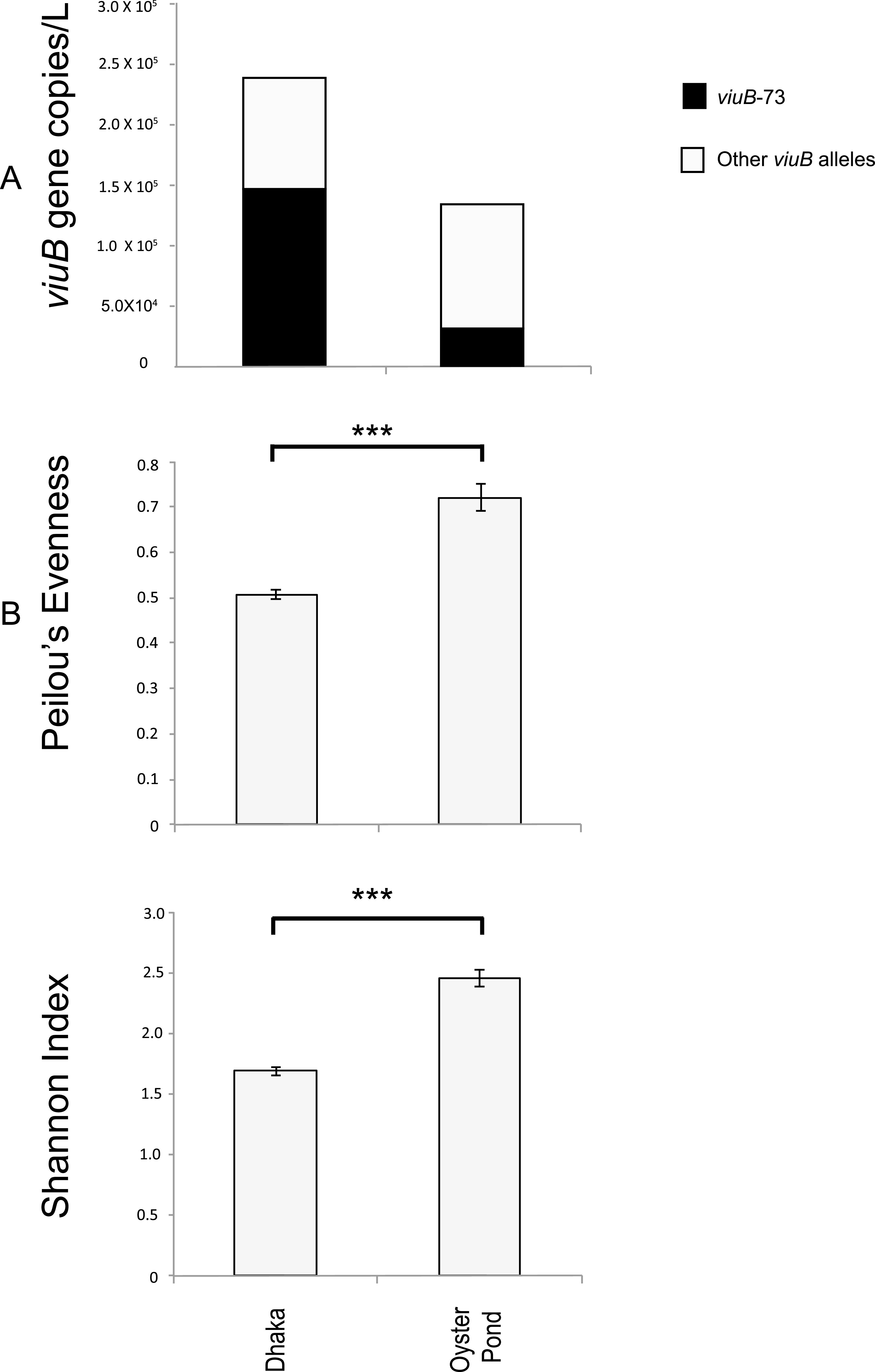
Abundance and diversity of Vibrio cholerae populations in two geographic locations, Dhaka and Oyster Pond. (A) Absolute average abundance of V. cholerae in two locations. *viuB* gene copy numbers were quantified from qPCR data; the average of *viuB* gene copies for all the samples in two locations was calculated and used as a proxy for the V. cholerae abundance. Total height of the bar represents total V. cholerae (*viuB*), the black segment represents *viuB*-73, and the clear segment represents other *viuB* alleles. (B) Evenness and diversity of the two V. cholerae populations measured by Peilou’s evenness and Shannon diversity indices based on analysis of *viuB* alleles. Statistical significance was measured by Kruskal-Wallis test; ***, statistically significant differences (Kruskal-Wallis, *P* < 0.01).

One of the main differences between the V. cholerae populations from Oyster Pond (Falmouth, MA, USA) and Dhaka (Bangladesh) is, unsurprisingly, the abundance of the pandemic-generating (PG) lineage, which includes strains responsible for the current 7th pandemic. Water samples were previously collected biweekly from seven different sites in the water bodies surrounding Dhaka city for nine continuous months (from June 2015 to March 2016) and were also collected from Oyster Pond over the summers of 2008 and 2009 in Cape Cod on the United States east coast (10). Here, we compare the V. cholerae populations from these two areas to gain insights into the differences between a region that experiences strong seasonal variation where cholera is not endemic and a tropical area where cholera is endemic. High-throughput sequencing of *viuB* marker gene amplicons was used to analyze the subspecies composition of V. cholerae in these two populations. Amplicons of this gene were annotated following a previously established scheme (9) in which diversity within the V. cholerae species is measured based on relative abundance and distribution of *viuB* alleles. Each allele represents a V. cholerae lineage, the diversity of which is roughly equivalent to that of a clonal complex as traditionally defined by multilocus sequence typing (9). A single *viuB* allele (*viuB*-73) is found to be uniquely associated with the PG lineage. Abundance and distribution of *viuB* alleles in samples collected from the two locations were estimated from *viuB* amplicon sequencing data normalized by quantitative data of *viuB* gene copy numbers determined by quantitative PCR (qPCR) (12). The total abundance of V. cholerae in the two locations varied significantly (Kruskal-Wallis test, *P* < 0.01), being almost twice as high on average in Dhaka (2.30 × 10^5^ gene copies/liter) than in Oyster Pond (1.25 × 10^5^ gene copies/liter) (Fig. 1A). However, when PG V. cholerae O1 (*viuB-*73) was excluded (quantified independently of other lineages using qPCR of the *rfb*O1 gene), average abundance was very similar in the two locations (Kruskal-Wallis, *P* < 0.01). The PG lineage was the predominant genotype in Dhaka, with an average abundance of 1.4 × 10^5^ *rfb*O1 gene copies/liter, whereas it was just a minor member of the population in Oyster Pond, with an average abundance of 1.5 × 10^4^ gene copies/liter (Fig. 1A). qPCR analysis confirmed that PG V. cholerae O1 present in the Oyster Pond population were nontoxigenic (CTX negative) as opposed to the vast majority of PG V. cholerae O1 in Dhaka being toxigenic (CTX positive) (12). Similarity percentage (SIMPER) analysis based on Bray-Curtis dissimilarity suggests that the allele most responsible for the overall dissimilarities between Dhaka and Oyster Pond is indeed *viuB*-73. This allele was predominant throughout the 9-month sampling period in Dhaka (11), constituting around 60% of the total V. cholerae population on average, whereas its presence was stochastic in Oyster Pond, constituting around 5% of the total population (9).

Population structure indices (diversity and evenness) were significantly lower in Dhaka than in Oyster Pond (Kruskal-Wallis test, *P* < 0.01) (Fig. 1B). This indicates a more stable and diverse V. cholerae community structure in the coastal location and a less diverse community dominated by fewer alleles in inland Bangladesh (Dhaka), likely because of the dominance of *viuB*-73 in that environment. Dhaka’s aquatic reservoirs therefore seem to harbor a V. cholerae community highly dominated by the PG lineage that is most likely to be affected substantially by human activity. It is one of the most densely populated megacities in the world and has a long history of suffering from recurring cholera (13). Maintenance of the cholera-causing genotype (PG) in the environment could be the driving factor to shape the overall population of V. cholerae in Dhaka. The reduction of intraspecies diversity by PG V. cholerae in Dhaka where cholera is endemic could be attributed to the potential selective advantage of colonizing the human gut (14), which would result in a constant output to water reservoirs. Type six secretion-mediated killing could also lead to the reduction of diversity, giving an advantage to PG V. cholerae in a resource-limited competitive environment, where PG V. cholerae is a superior competitor to other V. cholerae lineages under certain conditions. For example, in competition assays, PG V. cholerae has been shown to outcompete other lineages at a higher temperature (37°C), whereas at a lower temperature (25°C), other environmental lineages could outcompete it (40). Environmental conditions (i.e., the lower salinity seen in Dhaka [Table S1 in the supplemental material]) could also give an advantage to PG strains over others, as they have been shown to be more prevalent in lower-salinity environments relative to other lineages (9). In Oyster Pond, V. cholerae was present in substantially higher abundance in a coastal pond and lagoon compared to nearby ocean waters, indicating a likely ecological barrier (9). Overall, it is plausible that the combination of human population density and environmental factors creates the conditions for the maintenance of a natural V. cholerae population dominated by the PG lineage in Dhaka.

### A novel divergent lineage is endemic to inland Bangladesh.

**FIG 2:**
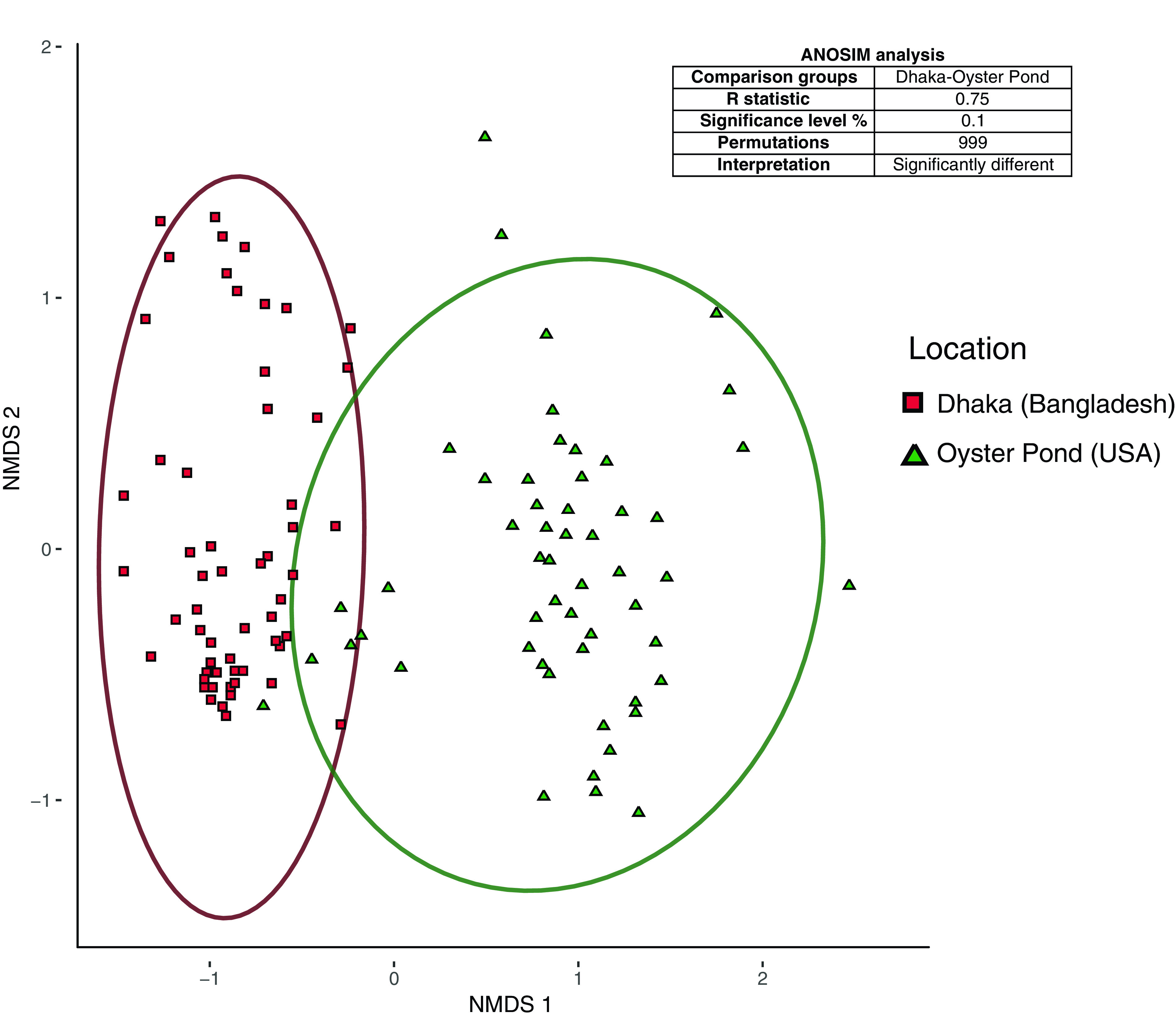
Nonmetric multidimensional scaling (NMDS) plot comparing beta diversities of Vibrio cholerae populations from two aquatic environments. Population compositions were compared using a Bray-Curtis dissimilarity matrix, with ellipses representing 95% confidence intervals. The data set was composed of *viuB* gene amplicon sequences normalized by qPCR copy numbers. The NMDS plot (stress 0.16) shows distinct clustering of samples from the two locations shown along the first two axes labeled as NMDS 1 and NMDS 2. Analyses of similarity (ANOSIM) results are displayed in the box inside the plot describing dissimilarity between pairs of samples from the two locations.

**FIG 3:**
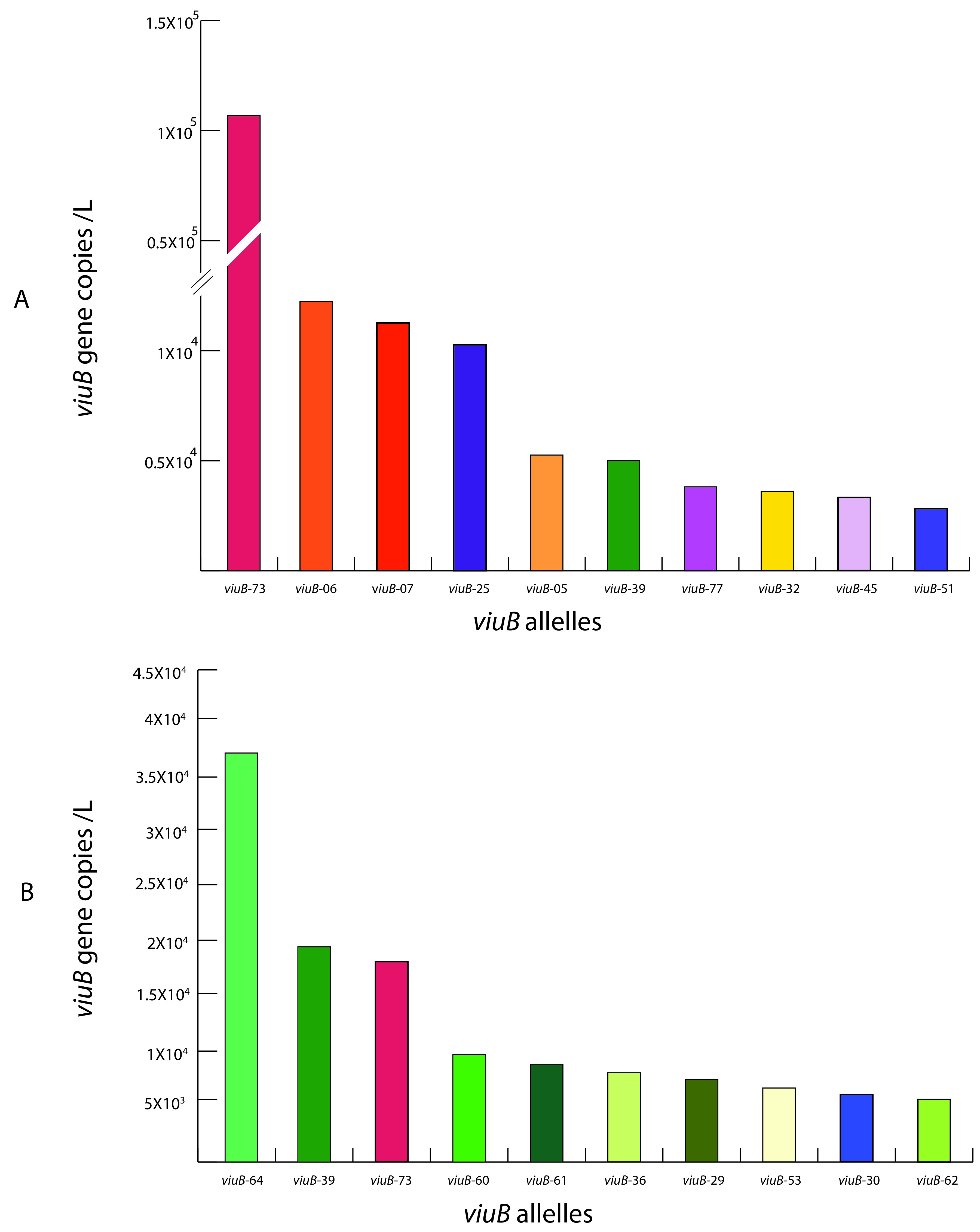
Abundance of the most prevalent *viuB* alleles at two locations: Dhaka (Bangladesh) (A) and Oyster Pond (USA) (B). Total *viuB* gene copy numbers were obtained by qPCR. Relative abundance of each allele was determined by amplicon sequencing. Specific colors were used for individual alleles to be consistent with a scheme described elsewhere (9). The 10 most abundant alleles for each location were selected for comparison between the two locations.

Besides the PG lineage, the population composition of V. cholerae sampled over 6 to 9 months was strikingly different in Dhaka and Oyster Pond. This was determined by using the abundance and distribution data of individual *viuB* alleles from the two locations. Nonmetric multidimensional scaling (NMDS) was performed to compare the two communities, and statistical significance of community structure dissimilarity was evaluated using the analysis of similarity (ANOSIM) with a Bray-Curtis distance matrix. In the NMDS plot, samples from Dhaka and Oyster Pond clustered separately, and community structure dissimilarity was statistically significant (ANOSIM *R* = 0.75, *P* < 0.01) (Fig. 2). Only two major alleles were shared between these locations from a total of 13 *viuB* alleles in Dhaka and 15 alleles in Oyster Pond (each individual allele constituting at least 1% of the V. cholerae population). The most abundant alleles in Dhaka after *viuB*-73 were *viuB-*06, *viuB-*07, *viuB-*25, and *viuB-*05 (Fig. 3). Of these four alleles, three are exclusively found in Dhaka (*viuB-*05, *viuB-*06, and *viuB-*07) and are of particular interest. Together, they composed ∼15% of the average Dhaka V. cholerae population and have been found to display higher abundance in sites surrounded by a high human population density and levels of pollution (16).

**FIG 4:**
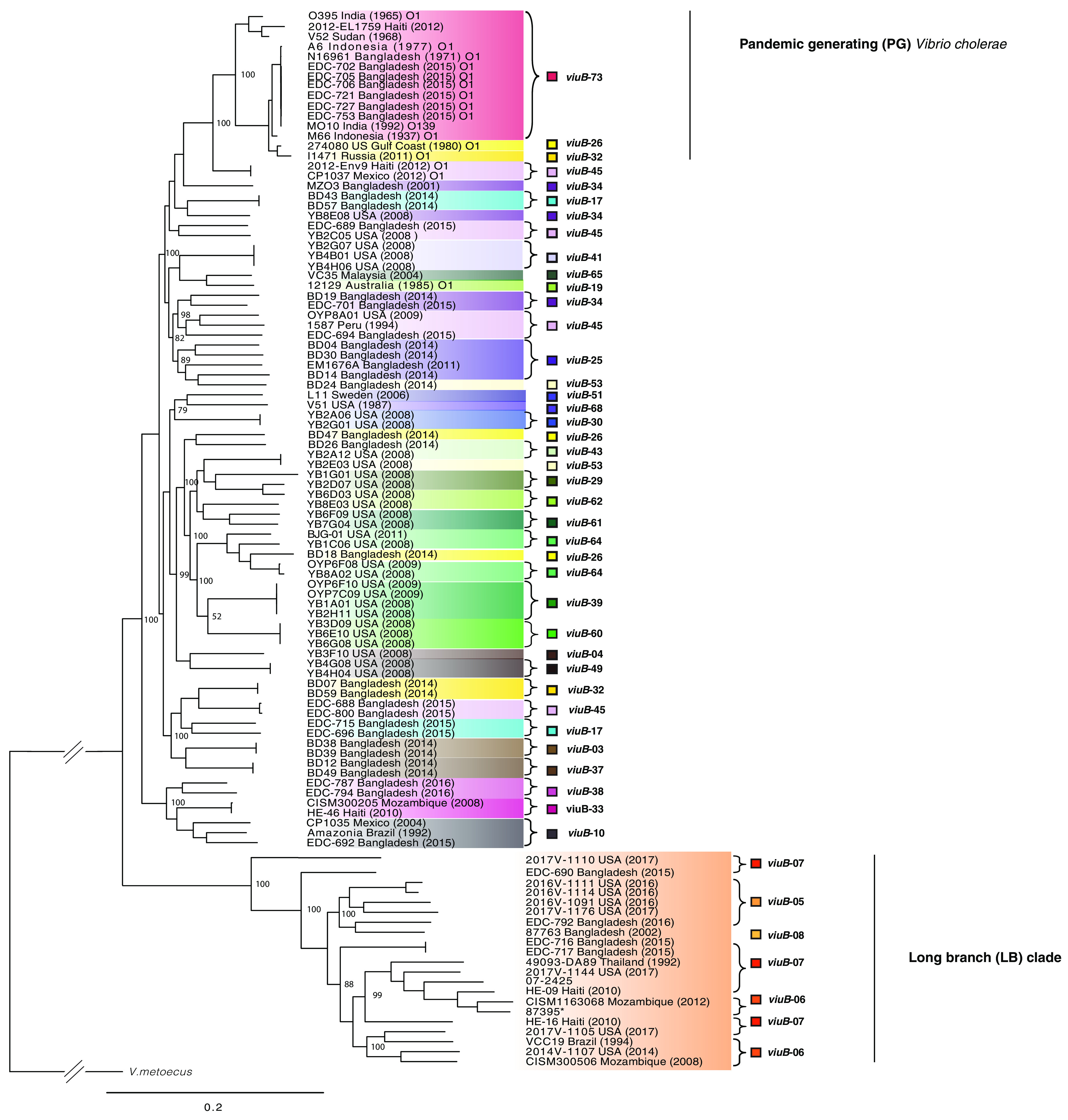
Whole-genome phylogeny of Vibrio cholerae strains found in Dhaka and Oyster Pond populations. The phylogenetic tree was inferred using Parsnp v1.2 (69) based on the reference genome of V. cholerae O1 El Tor N16961 and includes representative strains from other environments. Leaves of the tree were colored according to the *viuB* allele found in that particular genome. Statistical support of relevant nodes was estimated by bootstrap analysis (1,000 replicates, indicated as a percentage). The scale bar represents nucleotide substitutions per site.

To have more information on the lineages found in Dhaka, 23 V. cholerae strains isolated from the city during the study period were selected for whole-genome sequencing: 9 V. cholerae O1 isolates harboring the *viuB*-73 allele and 14 V. cholerae non-O1/O139 isolates displaying a diversity of *viuB* alleles. Four strains possessed *viuB* alleles 05, 06, 07, and 08 (EDC690, EDC716, EDC717, and EDC792) and were found to be part of a very long branch occupying a basal position in a global core genome phylogeny compared to the rest of the V. cholerae strains (hence termed long branch clade or LB) (Fig. 4). This phylogenetic group has not been described in any other studies, although other strains from public databases isolated from different parts of the world also belong to it (Table 1). Nine isolates were recovered from human clinical specimens across the United States and were reported to the CDC as part of the surveillance conducted under the Cholera and Other *Vibrio* Illness Surveillance (COVIS) program (17). Two more isolates originated from stool samples of diarrheal patients in Mozambique in 2008 (18), and one isolate was recovered from a diarrheal patient from Thailand in 1993 and was described as V. cholerae serogroup O155 (16). Seven additional isolates have been found to belong in the clade for a total of 23 as of August 2019 (Table 1).

**TABLE 1:** Demographic information of the *Vibrio paracholerae* sp. nov. and Vibrio cholerae isolates used in this study^*a*^

Isolates	Origin	Yr of isolation	Source	Phylogenetic group	Genome accession no.
N16961	Bangladesh	1971	Clin	PG Vibrio cholerae O1	AE003852.1
2010EL1786	Haiti	2010	Clin	PG Vibrio cholerae O1	NC_016445.1
EDC 721^*b*^	Bangladesh	2015	Env	PG Vibrio cholerae O1	WYCI00000000
MJ1236	Bangladesh	1994	Clin	PG Vibrio cholerae O1	NC_012668.1
MO10	India	1992	Clin	PG Vibrio cholerae O1	AAKF00000000
2740-80	USA	1980	Env	PG Vibrio cholerae O1	AAUT00000000
BX330286	Australia	1985	Env	PG Vibrio cholerae O1	ACIA00000000
O395	India	1965	Clin	PG Vibrio cholerae O1	CP001235, CP001236
95412	Mexico	1997	Env	PG Vibrio cholerae O1	NZ_KB662090.1
V52	Sudan	1968	Clin	PG Vibrio cholerae O37	AAKJ00000000
2012EL1759	Haiti	2012	Env	PG Vibrio cholerae O1	JNEW01000000
2012Env9	Haiti	2012	Env	Non-PG Vibrio cholerae O1	JSTH00000000.1
MZO-03	Bangladesh	2001	Clin	Non-PG Vibrio cholerae non-O1/O139	AAUU00000000
12129	Australia	1985	Env	Non-PG Vibrio cholerae O1	ACFQ00000000
1587	Peru	2000	Clin	Non-PG Vibrio cholerae non-O1/O139	AAUR00000000
CP1035	Mexico	2004	Clin	Non-PG Vibrio cholerae non-O1/O139	AJRM00000000
EDC 689^*b*^	Bangladesh	2015	Env	Non-PG Vibrio cholerae non-O1/O139	WYCR00000000
EDC 715^*b*^	Bangladesh	2016	Env	Non-PG Vibrio cholerae non-O1/O139	WYCJ00000000
EDC 800^*b*^	Bangladesh	2016	Env	Non-PG Vibrio cholerae non-O1/O139	WYCA00000000
EM1676A	Bangladesh	2011	Env	Non-PG Vibrio cholerae non-O1/O139	APFY00000000.1
2012ENV-92	Haiti	2012	Env	Non-PG Vibrio cholerae non-O1/O139	JSTJ00000000.1
HE 48	Haiti	2010	Env	Non-PG Vibrio cholerae non-O1/O139	AFOR00000000.1
VCC19	Brazil	1994	Env	*Vibrio paracholerae*	ATEV00000000.2
877163	Bangladesh	2002	Env	*Vibrio paracholerae*	LBNV00000000
EDC 792^*b*^	Bangladesh	2016	Env	*Vibrio paracholerae*	WYCC00000000
EDC 690^*b*^	Bangladesh	2015	Env	*Vibrio paracholerae*	WUWI00000000
EDC 716^*b*^	Bangladesh	2015	Env	*Vibrio paracholerae*	WYBZ00000000
EDC 717^*b*^	Bangladesh	2015	Env	*Vibrio paracholerae*	WYBY00000000
HE09	Haiti	2010	Env	*Vibrio paracholerae*	AFOP00000000.1
HE16	Haiti	2010	Env	*Vibrio paracholerae*	ALEB00000000.1
CISM300506	Mozambique	2008	Clin	*Vibrio paracholerae*	NZ_MWFL00000000
CISM1163068	Mozambique	2012	Clin	*Vibrio paracholerae*	NZ_MWFO01000150
49093 DA89	Thailand	1992	Clin	*Vibrio paracholerae*	JIDQ00000000.1
SIO	USA	2003	Waste water	*Vibrio paracholerae*	MIPJ01000076
2017V-1144	USA	2017	Stool	*Vibrio paracholerae*	QKKV00000000
2014V-1107	USA	2014	Stool	*Vibrio paracholerae*	QKKP00000000
2017V-1105	USA	2017	Wound	*Vibrio paracholerae*	QKKT00000000
2016V-1114	USA	2016	Stool	*Vibrio paracholerae*	QKKS00000000
2016V-1111	USA	2016	Stool	*Vibrio paracholerae*	QKKR00000000
2017V-1176	USA	2017	Animal feed	*Vibrio paracholerae*	QKKW00000000
2016V-1091	USA	2016	Stool	*Vibrio paracholerae*	QKKQ00000000
2017V-1110	USA	2017	Wound	*Vibrio paracholerae*	QKKU00000000
87395	UK	UK	UK	*Vibrio paracholerae*	APFL00000000.1
07-2425	UK	UK	UK	*Vibrio paracholerae*	QKKO00000000
NCTC30	Egypt	1916	Clin	*Vibrio paracholerae*	LS997868.1

a Clin, clinical; Env, environmental; PG, pandemic-generating; UK, unknown.

b Genomes sequenced for this study.

### A sister species to Vibrio cholerae?

Comparative genome analysis suggests that LB isolates could represent a new species, which would be the closest relative of V. cholerae described to date. Based on the genome sequences, G+C content of the strains belonging to the LB clade was 46% to 48.1%, falling within the known range of the genus *Vibrio*. Overall, a representative strain (EDC-792) of the clade shared 2,883 genes with type strains from both V. cholerae (N16961) and V. metoecus (OP3H) and 2,996 genes with V. cholerae alone (Fig. S1). The genomes of 22 LB isolates were compared with a set of V. cholerae strains containing the same number (*n* = 22) of representatives from both pandemic and nonpandemic lineages (Table 1). This comparison revealed that genetic distance between LB strains and V. cholerae fall below or at the threshold of the species cutoff values. Indeed, digital DNA-DNA hybridization (dDDH) values ranged from 82% to 100% within the LB clade and from 69% to 70% with V. cholerae, whereas average nucleotide identity (ANI) values ranged from 97% to 100% within the group and from 95% to 96% with V. cholerae strains, respectively. dDDH values are considered to be the gold standard for species designation, and a value of ≤70% presents as an indication that the tested organism belongs to a different species than the type strain(s) used as a reference (19). ANI has been proposed as an alternative genomic statistic to dDDH, and the cutoff values of 95% to 96% have been used for species delineation (20). In this case, all the strains from the LB clade had a dDDH value of ≤69% and an ANI value of ≤96% compared with the V. cholerae type strain N16961. In large-scale taxonomic studies, dDDH has been shown to overpower ANI values for the purpose of species designation (21, 22), suggesting that dDDH values should be given more importance when other evidence (phylogenetic, phenotypic, and ecological) support a novel species designation. Thus, according to the current species definition (23), the LB clade meets the genotypic criteria to qualify as a candidate for a novel species designation. It also meets the phylogenetic criteria, as it represents a well-defined, well-supported monophyletic clade in the core genome phylogeny (Fig. 4). Although the 16S rRNA gene has been used for taxonomic designations, it does not provide sufficient resolution to differentiate the closely related species in the V. cholerae clade (24, 25). For example, V. mimicus and V. metoecus, two species closely related to V. cholerae, show >99% 16S rRNA gene sequence identity with V. cholerae strains across the clade (7). We also performed a four-gene multilocus sequence analysis (MLSA), which has been used in studies describing several novel bacterial species in recent times (25, 26). Similar to the higher-resolution core genome phylogeny, the LB clade was well supported as a monophyletic cluster in the MLSA phylogenetic tree (Fig. S2). From an ecological perspective, lineages within the LB clade have shown distinct and cohesive abundance patterns in natural ecosystems of Dhaka, Bangladesh (11). Abundance and distribution of *viuB* alleles corresponding to the clade were found to be tightly correlated with each other, whereas they were negatively correlated with the abundance of the *viu*B allele corresponding to the dominant PG V. cholerae lineage (11).

**FIG 5:**
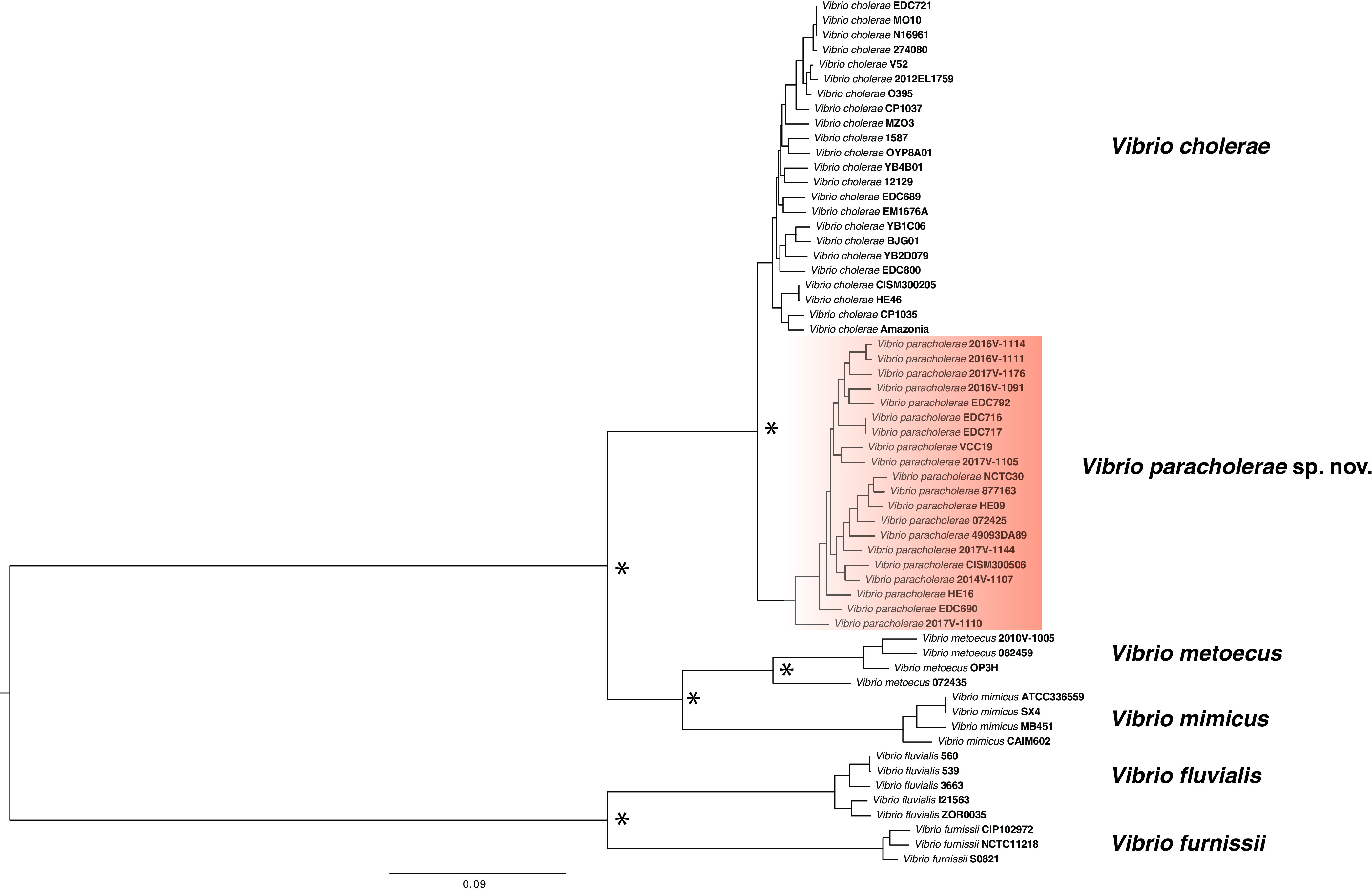
Whole-genome phylogenetic tree of *Vibrio paracholerae* along with its closest relatives. The maximum likelihood phylogenetic tree was constructed from the core genome alignment of ≈2.1 Mbp using the GTR gamma substitution model. Corresponding nodes with relevant bootstrap support over 70% from the 100 replicates were indicated with an asterisk (*). The scale bar represents nucleotide substitutions per site.

To confirm that the LB clade represents a novel species, its phenotypic traits were compared to those from the most closely related species V. cholerae and V. metoecus. Their ability to catabolize 190 different carbon sources and respond to 96 chemicals and antimicrobials was determined using Biolog phenotypic microarray (PM) plates. Four LB strains were examined, two of environmental origin from Bangladesh (EDC 690, EDC792) and two from clinical sources in the United States (2016V-1111, 2016V-1091). These strains were compared with four V. cholerae (N16961, V52, YB3B05, YB8E08) and four V. metoecus strains (082459, OP6B, OP4B, OP3H). Although the LB clade isolates resembled V. cholerae in most biochemical and growth characteristics, they clearly differed for some phenotypic characteristics (Table 2). All four LB strains tested could utilize α-cyclodextrin as a sole carbon source, whereas none of the tested V. cholerae strains could. Cyclodextrin utilization requires a specific category of amylases, which has not been reported in V. cholerae so far (27). *In silico* analysis revealed that LB strains possess a gene cluster (genes 03367 to 03379 in the NCTC30 genome NZ_LS997867) containing homologs of genes encoding cyclomaltodextrin glucanotransferase (*amyM*), ABC transporter MalK (*malK*), glycosidase MalE (*malE*), glucosamine *N*-acetyltransferase, cyclodextin-specific porin (*cycA*), cyclodextrin binding protein (*cycB*), cyclodextrin transport system permease (*malF*), cyclodextrin transport system permease (*malD/malG*), and neopullulanase (*nplT*) (Table 3). Only two (9%) V. cholerae strains in our data set (*n* = 22) and a similar percentage in the NCBI database possessed this cluster, whereas 100% of the LB clade strains (*n* = 22) harbored it. This gene cluster might be associated with the cyclodextrin degradation phenotype, as reported previously (28). LB isolates also differed clearly from V. cholerae in the utilization of pectin and monomethyl succinate (Table 2). In contrast with V. cholerae, 75% (3 out of 4) of LB isolates tested were found to be lacking the ability to utilize d-mannose, l-aspartic acid, and citric acid (Table 2). d-Mannose was found to be readily utilized by both V. cholerae and V. metoecus tested in this study, and previous literature reported that ∼80% of V. cholerae is capable of utilizing this sugar (3). The gene cluster encompassing *manP* to *manA* (VC1820 to VC1827 in the N16961 genome AE003852.1), including the well-known mannose-6 phosphate isomerase (*manA*) gene required for this process (29), was present in all the tested V. cholerae (*n* = 22) and V. metoecus (*n* = 22) strains, whereas it was found in only ∼40% (9/22) of LB strains (Table 3). Notably, three isolates that were unable to utilize mannose lack the gene cluster *manP*-*manA*, whereas the only isolate (2016V-1111) of the four tested isolates from the LB clade that could utilize mannose (Table 2) possesses the gene cluster in its genome. We could not find the genetic basis for the other phenotypic differences between LB isolates and V. cholerae. LB strains are similar to V. cholerae in *N*-acetyl-d-galactosamine d-glucuronic acid utilization tests and acetoin production, which differentiate both of the groups from V. metoecus (7). Resistance to 96 drugs or metals was also tested at different concentrations, and V. cholerae and LB isolates showed similar profiles in most tests, although three chemicals elicited differential responses by the two species. LB isolates were resistant to cadmium chloride, sodium selenite, and dichlofluanid in contrast to the sensitivity of the V. cholerae strains toward those chemicals (Table S2). Thus, phylogenetic, genotypic, phenotypic, and ecological data support the designation of a novel species for the LB clade. Very recently, genome sequencing efforts of a historical collection of isolates from cholera or cholera-like diseases have identified a strain isolated from a soldier convalescent in Egypt during the first World War (in 1916) as a divergent V. cholerae (30). This NCTC30 strain actually belongs to the LB clade found in this study (Fig. 5). Interestingly, NCTC30 was initially designated “Vibrio paracholerae” by Gardner and Venkatraman in 1935, and the disease caused was described as choleraic and termed as “paracholera” (15). To honor its history, we propose the name *Vibrio paracholerae* sp. nov. (EDC-792^T^) for this novel species. Inclusion of this novel species would revise the phylogeny of the V. cholerae species complex, making *V. paracholerae* sp. nov. the sister species to V. cholerae and V. metoecus as the sister species to V. mimicus (Fig. 5). This improved description of the species complex containing pandemic V. cholerae will provide a framework for understanding the emergence and evolution of human-pathogenic vibrios from their environmental common ancestor.

**TABLE 2:** Phenotypic traits differentiating *Vibrio paracholerae* sp. nov. from its closest relatives Vibrio cholerae and Vibrio metoecus

Phenotypic test^*a*^	*Vibrio paracholerae* sp. nov.^*b*^				Vibrio cholerae ^*c*^ ^,^ ^*d*^				Vibrio metoecus ^*d*^ ^,^ ^*e*^			
	1	2	3	4	5	6	7	8	9	10	11	12
α-Cyclodextrin	+	+	+	+	−	−	−	−	+	+	−	+
Pectin	+	+	+	+	−	−	−	−	+	+	+	+
Monomethyl succinate	−	−	−	−	+	+	+	+	−	−	−	−
d-Mannose	−	−	+	−	+	+	+	+	+	+	+	+
l-Aspartic acid	−	−	+	−	+	+	+	+	+	+	+	+
Citric acid	−	−	+	−	+	+	+	+	+	+	+	+
α-Keto glutaric acid	−	−	+	+	+	+	+	+	−	−	+	−
*N*-Acetyl-d-galactosamine	−	−	−	−	−	−	−	−	+	+	+	+
d-Glucuronic acid	−	−	−	−	−	−	−	−	+	+	+	+
Acetoin production	+	+	+	+	+	+	+	+	−	−	−	−

a +, growth/positive test result; −, no growth/negative test result.

b Tested strains: 1, EDC 792; 2, EDC 690; 3, 2016V-1111; 4, 2016V-1091.

c Tested strains: 5, N16961; 6, V52; 7, YB3B05; 8, YB8E08.

d Results for V. cholerae and V. metoecus strains were obtained from Kirchberger et al. (7).

e Tested strains: 9, 082459; 10, OP6B; 11, OP4B; 12, OP3H.

**TABLE 3:** Major genetic traits differentiating *Vibrio paracholerae* sp. nov. from its closest relatives Vibrio cholerae and Vibrio metoecus

Genomic island/gene cluster	Genomic position in reference genomes^*a*^		Present in % strains^*b*^			Putative function	Reference
	N16961 (AE003852.1) locus	NCTC30 (LS997868.1) locus	VC (*n* = 22)	VP (*n* = 22)	VM (*n* = 22)		
RND efflux pump gene cluster	Absent	818–823	0	90	0	Resistance to antimicrobials and heavy metals	This study
GI-66	Absent	1923–1927	0	100	0	Iron regulation	43
Cyclo-maltodextrin operon	Absent	3367–3375	9	100	68	Cyclodextrin utilization	27
VCA1102-1111 (N16961)	VCA_1102–VCA_1111	Absent	100	0	100	Fatty acid biosynthesis, heme biliverdin, thermostable hemolysin	This study
Tor operon	VC_1692–VC_1694, VC_1719–VC_1720	Absent	100	0	100	Virulence gene regulation	31
Glutathione-regulated potassium pump	VC_2606–VC_2607	Absent	100	0	100	Potassium regulation	This study
Beta lactamase	Absent	3210	0	60	0	Resistance to β-lactams	30
*manA*-*manP*	VC_1820–VC_1827	1534–1543	68	22	100	Utilization of mannose	29

a Reference genomes N16961 (V. cholerae) and NCTC30 (*V. paracholerae* sp. nov.) were used for determining locus positions of the gene clusters.

b VC, Vibrio cholerae; VM, Vibrio metoecus; VP, Vibrio paracholerae sp. nov.

### A potential threat to humans?

To be a successful disease-causing agent to humans, a bacterial pathogen of aquatic origin needs to have the ability to survive in the environment and colonize the human body. In Dhaka, where cholera is endemic, *V. paracholerae* sp. nov. has been found to exist abundantly in local water reservoirs. In one particular site, the number even surpassed that of PG V. cholerae, which was otherwise the most predominant lineage found in Dhaka (11). That site (Kamrangirchar) happened to be the most densely populated area among those sampled, indicating a possible link between human population and the prevalence of *V. paracholerae* sp. nov. Overall, ecological analysis of the population in Dhaka suggests that the environmental abundance and distribution of the lineages corresponding to *V. paracholerae* sp. nov. strains are correlated with human population density (11). Although data from other locations are required to confirm this correlation, this observation raises the possibility of adaptation to the human gut as an alternate niche and an important factor in the ecology of this novel species. Association of the members of the species with cholera-like cases, such as in the case of the historical strain NCTC30, and isolation from clinical/human stool samples from different parts of the world (Table 1; Table S4) would suggest their pathogenic potential to humans. To assess this potential, *V. paracholerae* sp. nov. strains were screened for the presence of known virulence-related genes and islands often found in V. cholerae (Table S3).

*V. paracholerae* sp. nov. strains lack CTX, VPI-1, and VPI-2 (Table S3; Fig. S3), three major elements known to be essential for V. cholerae to cause cholera (2). They also lack a cluster of genes (VC1692, VC1694, VC1719, and VC1720 in the N16961 genome) encoding proteins for the “Tor operon” required for trimethylamine *N*-oxide respiration in V. cholerae. The genes in this operon have been shown to be crucial for cholera toxin production, cytotoxicity, and intestinal colonization of V. cholerae in an infant mouse model (31). The Tor operon was found in 100% of V. cholerae (*n* = 22) and *V. metoecus* (*n* = 4) strains in our data set, which indicates that it was likely lost in the *V. paracholerae* sp. nov. phylogenetic branch. All the *V. paracholerae* sp. nov. strains in our data set possessed the RTX toxin gene cluster (Table S3), a virulence factor for V. cholerae known to have a role in interactions with eukaryotes (32). Interestingly, four *V. paracholerae* sp. nov. strains (22%) (including NCTC30) possess type three secretion system (T3SS) genes (Table S3), an established virulence factor for nonpandemic V. cholerae (33). The historical strain NCTC30, which was isolated from a choleraic patient, contains a T3SS genetically more similar to a V. parahaemolyticus T3SS than that of one found in a subset of V. cholerae (acquired within a VPI-2 insertion site locus of PG V. cholerae) (30). The region of *V. paracholerae* sp. nov. strains containing this T3SS is 97% identical at the nucleotide sequence level to the T3SS island found in V. parahaemolyticus, highlighting a recent acquisition from this human-pathogenic species (Fig. S4). However, NCTC30 does not have the mobile integrative conjugative element (ICE) SXT/RC39, whereas four *V. paracholerae* sp. nov. strains contain this element in their genomes (Table S3). SXT/RC39-related ICEs are found in most PG V. cholerae strains since 2001 and are believed to be involved in improved fitness in 7th pandemic El Tor V. cholerae (34, 35). ICEs found in *V. paracholerae* sp. nov. strains showed 95.7% to 96.4% nucleotide sequence identity in the conserved homologous regions to the first reported SXT element in V. cholerae SXT^MO10^, originally detected in V. cholerae O139 strain MO10 (36). However, ICEs found in *V. paracholerae* sp. nov. strains were more related to ICEVchMex1, which was first reported to be present in an environmental V. cholerae non-O1/O139 strain (36) based on the nucleotide sequence of the integrase (*int*) gene (Fig. S7). Additionally, 50% of the *V. paracholerae* sp. nov. strains also contained genes for the recently discovered cholix toxin, thought to be an important virulence factor for V. cholerae (37). Recently, a novel island has been found in the VPI-1 insertion site, designated GI*Vch*S12, which contains CRISPR-Cas and type 6 secretion system (T6SS) modules (38). CRISPR-Cas is a defense mechanism that bacteria can utilize against unwanted lateral gene transfer by recognizing foreign DNA and cleaving it. CRISPR-Cas modules are usually unevenly distributed across taxa, but can potentially provide a fitness advantage to the bacteria in a mixed population (38, 39). Seven out of 22 strains (∼31%) contain a version of the GI*Vch*S12 island in the region to the VPI-1 insertion site of PG V. cholerae (Fig. S5). The T6SS operon of Vibrio cholerae contains diverse arrays of toxic effector and cognate immunity genes, which are thought to play an important role in the environmental lifestyle and adaptation to the human host (40). The functional T6SS genetic element corresponds to three loci across the V. cholerae genome, termed aux-1, aux-2, and large cluster (41). Like V. cholerae and its close relatives *V. mimicus* and *V. metoecus* (40), *V. paracholerae* sp. nov. possesses all three chromosomally encoded T6SS loci and shares its effector and immunity gene pool with these species (Table S5). However, for a similar phylogenetic diversity, the diversity of the T6SS effector-immunity loci is much less in *V. paracholerae* sp. nov. than that in V. cholerae (40, 42). This surprising lack of diversity could represent an ecological adaption in *V. paracholerae*, which could be deriving a fitness advantage in specific niches from this restricted set of T6SS effector and immunity proteins.

Apart from the known virulence genes usually found in V. cholerae, gene content analysis revealed a few species-specific genetic traits in *V. paracholerae* sp. nov. Two genes were present in all 22 *V. paracholerae* sp. nov. strains, with no homolog found in any V. cholerae strains. These two genes encode a LysR family transcriptional regulator (WP_001924807.1) and an HAD-IB family hydrolase (WP_071179638.1). Both of these genes are part of a previously reported genomic island (GI-66) found in Vibrio albensis (43). This genomic island contains iron-related regulatory genes that can be significant in the regulation of iron scavenging in this group of organisms. Iron acquisition is thought to be an important aspect for the regulation of virulence as well as host selectivity/specificity (44). Most (90%) of *V. paracholerae* sp. nov. strains also harbored a novel resistance-nodulation-division (RND) efflux pump (Table 3) thought to be critical for intrinsic and induced antimicrobial resistance, virulence gene expression, colonization in an animal host, and environmental regulation of stress response (45). Efflux pumps have been proposed to be important for expelling bile out of the cell, and the resulting bile resistance would be key to overcoming this challenge inside the human gut (46). RND efflux pumps have specifically been found to confer increased bile resistance in other Gram-negative bacteria (47). The novel RND gene cluster is absent in both V. cholerae and *V. metoecus*, but homologs have been found in the halophilic bacteria V. cincinnatiensis (48) and a bile-associated isolate of V. fluvialis (49). Other than efflux pumps, cholera toxin transcriptional activator (ToxR) and outer membrane protein TolC have also been proposed to be crucial for bile resistance, and, like V. cholerae strains, all the *V. paracholerae* sp. nov. strains possess both genes. All these factors make *V. paracholerae* sp. nov. a potential candidate for a species associated with the human gut microbial population and underscore the importance of studying their biology in greater detail.

### Interaction of *Vibrio paracholerae* sp. nov. with pandemic Vibrio cholerae could have impacted the ecology and evolution of both species.

Horizontal gene transfer (HGT) among species sharing an ecological niche can have a major impact on their evolution (50). As *V. paracholerae* sp. nov. coexists with V. cholerae in natural ecosystems (at least in Dhaka), it is expected that HGT could take place between these two groups. To assess the propensity of interspecies HGT, potential gene transfer events between two groups (V. cholerae and *V. paracholerae* sp. nov.) were inferred based on phylogenetic congruence of individual genes. Maximum likelihood (ML) trees were constructed for each of the core and accessory gene families present in at least two strains from each group. A gene transfer was hypothesized if a member of a group clustered with members of the other group in a clade, and the gene tree could not be partitioned into perfect clades, which must consist of all members from the same group and only of that group (8, 51). In our groups of 22 V. cholerae and 22 *V. paracholerae* sp. nov. strains, 216 HGT events were hypothesized, involving 82 gene families from V. cholerae to *V. paracholerae* sp. nov., but only 62 events from *V. paracholerae* sp. nov. to V. cholerae involving 33 gene families. All of the core genes transferred from *V. paracholerae* sp. nov. to V. cholerae were acquired by strains outside the PG group.

In the case of accessory genes, we could infer 82 potential transfer events from V. cholerae to *V. paracholerae* sp. nov. and 54 events from *V. paracholerae* sp. nov. to V. cholerae. Only four events involved strains belonging to the PG clade. Thus, gene transfer directionality was biased from V. cholerae to *V. paracholerae* sp. nov., *V. paracholerae* sp. nov. being the recipient of HGT in most cases. A lower rate of HGT toward V. cholerae was previously reported with the cooccurring *V. metoecus*, which has a lower abundance in the environment (8). This gene transfer bias could be attributed to the dominance of V. cholerae in an area where cholera is endemic, as it is generally more abundant than *V. paracholerae* sp. nov. and, therefore, more likely to be a DNA donor (12). Among the accessory genes transferred from V. cholerae to *V. paracholerae* sp. nov., there were proteins related to O antigen synthesis, the type six secretion system, iron regulation, chaperone and multidrug resistance, and putative metabolic functions. There are examples of a single gene or even a small set of nucleotides within a gene acquired via HGT impacting the ecology and pathogenicity of *Vibrio* species (50, 52). Thus, the HGT events in *V. paracholerae* sp. nov. underscore the possibility for species coexisting with PG V. cholerae to acquire virulence and fitness-related genes to become pathogenic to humans and/or develop novel ecological traits.

**FIG 6:**
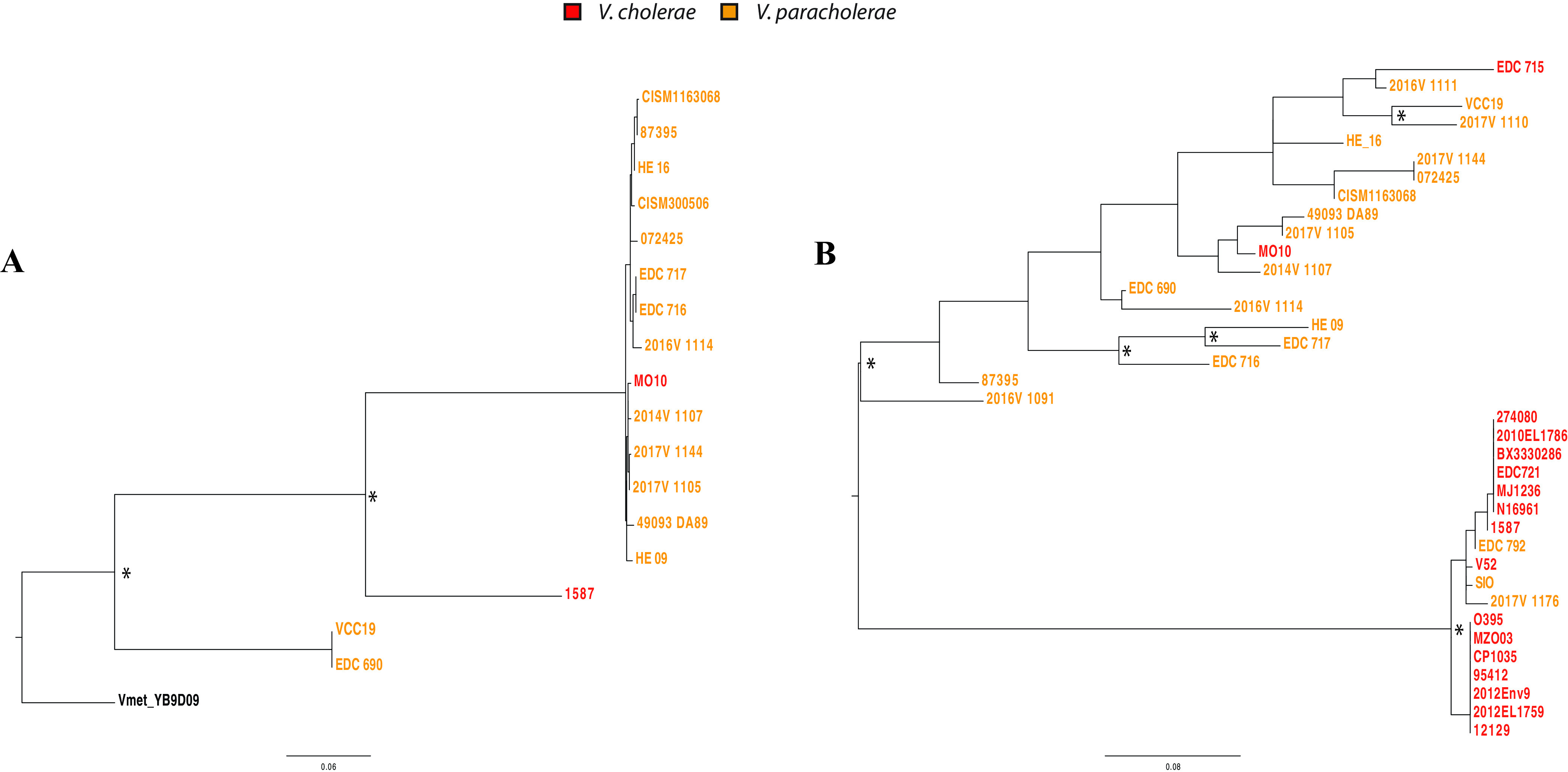
Phylogenetic tree of O antigen cluster genes found in *Vibrio paracholerae* and Vibrio cholerae. Maximum likelihood trees were constructed using a 705-bp nucleotide alignment of the gene encoding UDP-glucose 4-epimerase (A) and a 564-bp alignment of the gene encoding UDP-*N* acetylgalactosaminyltransferase (B). Nodes with relevant bootstrap support over 70% of 100 replicates are indicated with an asterisk (*). The scale bar represents nucleotide substitutions per site.

Gene transfer events have led to the rise of virulent V. cholerae before, the most striking example being the rise of V. cholerae O139. The latter emerged in Bangladesh and India in 1992 and has been hypothesized to have originated via genetic recombination of the O antigen region from a serogroup O22 strain to a serogroup O1 El Tor strain (53). After its emergence, V. cholerae O139 remained an important cause of widespread cholera epidemics in that region until 2004, along with V. cholerae O1 El Tor (53). Interestingly, among the accessory genes inferred as subject to HGT from *V. paracholerae* sp. nov. to V. cholerae, genes encoding UDP-glucose 4-epimerase (EC 5.1.3.2) and UDP-*N*-acetylgalactosaminyltransferase appear to be a transfer in an ancestor of O139 strain MO10 from the *V. paracholerae* sp. nov. clade (Fig. 6). Both of these genes are involved in O antigen biosynthesis and could be of significance in the emergence and evolution of V. cholerae O139 as a human pathogen and pandemic agent. Even though it will require further investigation to find out how and to what extent *V. paracholerae* sp. nov. as a species contributed to the emergence and evolution of V. cholerae O139, these transfer events could be considered examples of how interaction of this close relative with V. cholerae could impact the epidemiology of cholera. The O antigen region is a highly variable region in terms of gene content. Previously, O22 strains have been hypothesized as the source of the O139 regions not found is V. cholerae O1 El Tor. However, there are parts of the O antigen region in V. cholerae reference O139 strain MO10 (designated region IV by Yamasaki et al.) for which the origin could not be attributed to either O22 or V. cholerae O1 (54). Recently, closely related homologs of genes in region IV of V. cholerae O139 strains were found in V. fujianensis (55). This indicates that the origin of the MO10 O antigen region is most likely a result of multiple recombination events from various origins, forming the mosaic O antigen-encoding region in V. cholerae O139. *V. paracholerae* sp. nov. strains contain at least nine genes found in the V. cholerae O139 reference strain MO10 that are not found in V. cholerae O1 (Fig. S6). These observations suggest that *V. paracholerae* sp. nov. could have been an important player of the gene transfer dynamics that gave rise to the second pathogenic serogroup of V. cholerae.

### Conclusions.

Culture-independent analysis below the species level in inland locations where cholera is endemic and in coastal locations where cholera is not endemic in distinct geographic settings identified differences in the population structures present in these environments. It revealed that human influences are likely to be a major factor shaping communities of that species in areas where cholera is endemic. In urban tropical Dhaka, found in inland Bangladesh, PG V. cholerae was abundant and continuously present, but was accompanied by members of a related, but phylogenetically distinct, clade, which could represent a novel species. The abundance of this putative species, “*Vibrio paracholerae* sp. nov.,” in Dhaka and its absence from Oyster Pond on the United States east coast indicates that it is not a ubiquitous member of aquatic communities. In addition to those identified from the COVIS program in the United States and from Mozambique and Thailand, a number of strains of *Vibrio* spp. have been very recently isolated from clinical cases in China and Korea, which would belong to this species according to the genome sequence similarities they share with strains analyzed here (Table S4). An indirect association of their abundance with human population density indicates that they could be adapted to the human gut in areas where cholera is endemic (11). They could therefore occasionally become pathogenic by acquiring pathogenicity gene clusters or cause opportunistic infections in vulnerable individuals. Its history, biology, genetic traits, and coexistence with a pathogenic sister species make it a risk as an emerging human pathogen. Its potential contribution to the evolution of new pathogenic variants of V. cholerae (such as PG lineage O139) and likely influence on their population structure highlights the importance of studying this novel species in the context of a globally distributed infectious disease.

### Taxonomy.

*Vibrio paracholerae* (pa.ra.chol.er.ae. Gr. prep. *para*, alongside; L. gen. f. n. *cholerae*, bilious disease, referring to the isolation of the type strain alongside Vibrio cholerae, the causative agent of cholera). A Gram-negative, oxidase-positive, curved rod-shaped bacterium, roughly 1.25 to 2 mm in length and 0.4 mm in width. Exhibits motility by means of a single polar flagellum. Growth is observed at 30°C with salt concentrations in the range of 0% to 6.0% NaCl; no growth occurs in the presence of 10% NaCl. The ability to utilize α-cyclodextrin and pectin as well as the lack of ability to utilize monomethyl succinate differentiates the species from its closest relative V. cholerae (99% identity of the 16S rRNA gene). Forms yellow circular colonies on thiosulfate citrate bile salts sucrose (TCBS) agar (similar to those formed by *V. cholerae*) and circular colonies of creamy white color on tryptic soy broth (TSB) agar. Positive for carbon utilization from d-glucose, d-fructose, sucrose, maltose, d-galactose, maltotriose, d-mannitol, d-trehalose, d-glucose-6-phosphate, *N*-acetyl-d-glucosamine, glycerol, succinic acid, l-lactic acid, l-glutamic acid, fumaric acid, acetic acid, l-proline, d-alanine, l-asparagine, 2-deoxyadenosine, adenosine, inosine and l-serine, dextrin, gelatin, glycogen, d-glucosamine, and d-lactic acid methyl ester. Variation of response observed between strains in utilization of d-mannose, d-cellobiose, d-fructose-6-phosphate, d-psicose, α-d-lactose, l-aspartic acid, d-glucuronic acid, d-gluconic acid, d,l-α-glycerol phosphate, d,l-malic acid, d-ribose, Tween-20, thymidine, α-keto-glutaric acid, Tween-40, Tween-80, α-keto-butyric acid, uridine, l-glutamine, α-hydroxy-glutaric acid-γ-lactone, β-methyl-d-glucoside, citric acid, l-threonine, l-alanine, l-alanyl-glycine, glycine, histidine, methyl pyruvate, d-malic acid, glycyl-l-proline, and pyruvic acid. The proposed type strain EDC-792 was isolated from environmental water in Dhaka, Bangladesh, in 2016; the strain displays all the properties given above for the species. The proposed type strain (EDC-792 = DSM 112460^T^ = CCUG 75319^T^) shows 95.6% ANI and 69% dDDH, respectively, to the type strain of the closest sister species, Vibrio cholerae (N16961). The oldest isolate of the species, NCTC30, described by Dorman et al. (30), is likely to have some phenotype-changing mutations that urged for a new type strain with typical properties present in all the known strains of the species.

## MATERIALS AND METHODS

### Sample collection and processing.

Environmental water samples were collected every 2 weeks between June 2015 and March 2016 from seven points along the water bodies surrounding Dhaka city, which is located in the central part of Bangladesh (23.8103°N, 90.4125°E). One-time water samples were collected from two natural coastal water bodies in Mathbaria (22.2920°N, 89.9580°E) and Kuakata (21.8210°N, 90.1214°E), which are geographically adjacent to the coast of the Bay of Bengal and approximately 200 km and 250 km southwest of Dhaka, respectively. One liter of water was collected from each site in sterile Nalgene bottles placed in an insulated plastic box and transported at ambient air temperature from the site of collection to the central laboratory of the International Center for Diarrheal Disease Research, Bangladesh (icddr,b), in Dhaka. Oyster Pond sampling was performed at the same spots and at approximately the same time of day in the pond and the nearby lagoon connected to the ocean in monthly intervals from June to October in 2008 and 2009 as described in Kirchberger et al. (10). Fifty liters of water was filtered through 0.22-μm Sterivex filters (Mo Bio Laboratories Inc., Carlsbad, CA, USA) for the collection of biomass. Genomic DNA was extracted from the biomass using the protocol described by Wright et al. (56).

### Isolation and identification of isolates.

Bacterial isolates were recovered as described elsewhere (57). Briefly, water samples were enriched in alkaline peptone water (APW; Difco Laboratories, Detroit, MI) at 37°C for 6 to 8 h before plating. About 5 μl of enriched APW broth was streaked using an inoculating loop onto both thiosulfate citrate bile salts sucrose (TCBS) and taurocholate tellurite gelatin agar (TTGA) and incubated at 37°C for 18 to 24 h. Colonies with the characteristic appearance of V. cholerae were confirmed by standard biochemical and serological tests (and, in the case of the latter, by testing with polyvalent and monoclonal antibodies specific for V. cholerae O1 or O139) and, finally, by PCR.

### Phenotypic tests.

For the comparison of phenotypic characteristics, Biolog phenotypic microarray plates PM1, PM2A, PM14A, PM16A, and PM18C were used (58). Overnight-cultured bacterial colonies were inoculated into Biolog IF-0a base medium to reach 85% turbidity, and a 1:200 dilution was aliquoted into IF-10b medium supplemented with dye mix A, as indicated by the manufacturer’s instructions. The mixture was then added into wells of Biolog PM1 and PM2A plates containing various carbon sources and PM14A, PM16A, and PM18C plates containing substrates of various antimicrobials and heavy metal salts. The incubation and monitoring of the growth of inocula were done for 96 h in the presence of a sole carbon source or the heavy metals; growth causes reduction of the dye, resulting in purple color formation.

### Quantitative PCR.

Estimation of Vibrio cholerae number was done using qPCR following the protocol described elsewhere (12). The target probe for *viuB*, 5′-/56-FAM/TCATTTGGC/ZEN/CAGAGCATAAACCGGT/3IABkFQ/-3′, the forward primer, 5′-TCGGTATTGTCTAACGGTAT-3′, and the reverse primer, 5′-CGATTCGTGAGGGTGATA-3′, were used. The volume of the PCR reaction was 10 μl and contained 5 μl of 2× Dynamite qPCR master mix (MBSU, University of Alberta, Edmonton, Canada), 1 μl each of 500 nM primer-250 nM probe mix, 1 μl of molecular-grade water, and 2 μl of DNA template. Real-time quantitative PCR was performed under the following conditions: initial primer activation at 95°C for 2 min followed by 40 cycles of 95°C for 15 s and 60°C for 1 min in an Illumina Eco real-time PCR system.

### Amplicon sequencing.

Amplicon sequencing of the *viuB* gene was performed following the method described elsewhere (9). To amplify 293 bp of the *viuB* region from DNA extracted from water samples, a touchdown PCR was performed using 0.5 μl each of 10 pmol forward and reverse primers (for *viuB*: *viuB*2f 5′-CCGTTAGACAATACCGAGCAC-3′ and *viuB*5r 5′-TTAGGATCGCGCACTAACCAC-3′), 0.4 μl of 10 mM dNTP mix (ThermoFisher), 0.4 μl of Phire Hot Start II DNA polymerase (ThermoFisher), 0.5 μl of molecular biology-grade bovine serum albumin (20 mg/ml, New England Biolabs), 5 μl of 5× Phire buffer, and 2 μl of template DNA. The PCR was performed as follows: initial denaturation at 98°C for 4 min, followed by 10 cycles of denaturation at 98°C for 10 s, annealing at 60°C for 6 s (reduced by 1°C per cycle), and extension at 72°C for 1 s, followed by 23 cycles of denaturation at 98°C for 10 s, annealing at 50°C for 6 s (reduced by 1°C per cycle), and extension at 72°C for 1 s, and a final extension at 72°C for 1 min. In preparation for sequencing, dual-indexed sequences were tagged using indices developed by Kozich et al. (59) as follows: 2 μl of preceding *viuB* PCR amplification reaction was used as the template for tagging PCR; initial denaturation at 98°C for 30 s, followed by two cycles of denaturation at 98°C for 10 s, annealing at 55°C for 6 s, and extension at 72°C for 1 s, and final extension at 72°C for 1 min. Eight tagging reactions were performed for each sample, and products were pooled and run on a 2% agarose gel in 1× Tris-acetate-EDTA buffer. The appropriate bands (428 bp) were cut out of the gel. PCR products were then purified using the Wizard SV gel and PCR clean-up system (Promega) according to the manufacturer’s instructions. Concentration of clean PCR products was then measured using a Qubit fluorometer (ThermoFisher) with a Qubit double-stranded DNA high-sensitivity assay kit (ThermoFisher), and products were pooled in equal concentrations (>10 ng/μl). The pooled samples were then concentrated using a Wizard SV gel and PCR clean-up system (Promega). Quality control of the pooled and concentrated sample was done using an Agilent 2100 Bioanalyzer. Sequencing was performed using Illumina MiSeq technology with a v3 (600 cycles) reagent kit.

### Amplicon sequence analysis.

Demultiplexed raw reads from the sequencing run were processed in R (60) using the DADA2 pipeline 1.4.0 (61). First, 10 bp of forward and reverse reads were trimmed, and reads with a maximum expected error rate of >1 were discarded. Chimera detection implemented in DADA2 was then performed on pooled samples. To account for the possibility of real chimeras between protein-coding genes from closely related organisms (due to recombination or homoplastic mutations), chimeras were compared with a reference data set of *viuB* alleles found in 782 sequenced V. cholerae genomes (obtained from GenBank). Only *viuB* alleles composed of more than 1,000 reads found in multiple samples (with an average of 100,000 reads per sample) were considered for further analysis. Samples were rarefied to the level of the sample with the lowest reads using mothur 1.39.5 (62), and further analysis was performed in R, with statistical tests and distance calculations performed using the VEGAN 2.4-6 package (63). Bray-Curtis similarity was calculated based on relative read abundance of each allele in different samples in the Primer-E software suite and used for similarity percentage (SIMPER) and nonmetric multidimensional scaling (NMDS) analysis.

### Whole-genome sequencing and core genome phylogeny.

The genomes of 23 strains from Dhaka belonging to various *viuB* genotypes were chosen for whole-genome sequencing as described in Orata et al. (8). Sequencing libraries were prepared from genomic DNA using the Nextera XT DNA library preparation kit (Illumina, San Diego, CA, USA) and sequenced using Illumina MiSeq sequencing platforms (2 × 250-bp paired-end reads). Quality control and *de novo* assembly of the reads were done using default parameters in CLC Genomics workbench 7 (Qiagen). Whole-genome alignment was performed using Mugsy v1.2.3 (64) with default parameters, and a maximum likelihood tree was built from this alignment using RaxML v8 (65) under the general time reversible (GTR) gamma model with 100 bootstrap replicates. Additional V. cholerae genomes were downloaded from GenBank. The maximum likelihood phylogenomic tree was constructed from the alignment of locally colinear blocks (2,094,734 bp) using a GTR gamma substitution model with 100 bootstrap replicates.

### Comparative genomic analysis.

The genome sequences were annotated with RAST 2.0 (66). Genomic distances were calculated in Geneious (67). Core and accessory genes were determined with BPGA, finding orthologous protein-coding genes clustered into families based on a 30% amino acid sequence identity (68). Group-specific genes were clustered using a custom-made Python program. BLAST atlas of the genomes and genomic islands was carried out using GView server (https://server.gview.ca/). Effector and immunity genes in type 6 secretion system loci were typed as previously described in Kirchberger et al. (42).

### Data availability.

All previously sequenced bacterial genomes and genomes sequenced in this study are available from the NCBI GenBank and the PubMLST databases. Table 1 and Table S5 in the supplemental material list all the accession numbers for the genomes used in this study. In addition, all genome sequences and relevant genomic and epidemiological data related to the isolates used are publicly available on PubMLST (https://pubmlst.org/vcholerae/).
