# Supplementary material for: Population Analysis of Vibrio cholerae in Aquatic Reservoirs Reveals a Novel Sister Species (Vibrio paracholerae sp. nov.) with a History of Association with Humans
Source: Appl Environ Microbiol. 2021 Aug 11;87(17):e00422-21. doi: 10.1128/AEM.00422-21 (PMC8357300; doi:10.1128/AEM.00422-21)
Supplement: Supplemental file 1 — Figures S1 to S7, Tables S1 to S5. Download AEM00422-21_Supp_1_seq13.pdf, PDF file, 2.8 MB [file aem00422-21_supp_1_seq13.pdf]

Supplementary material:

Supplementary tables and figures:

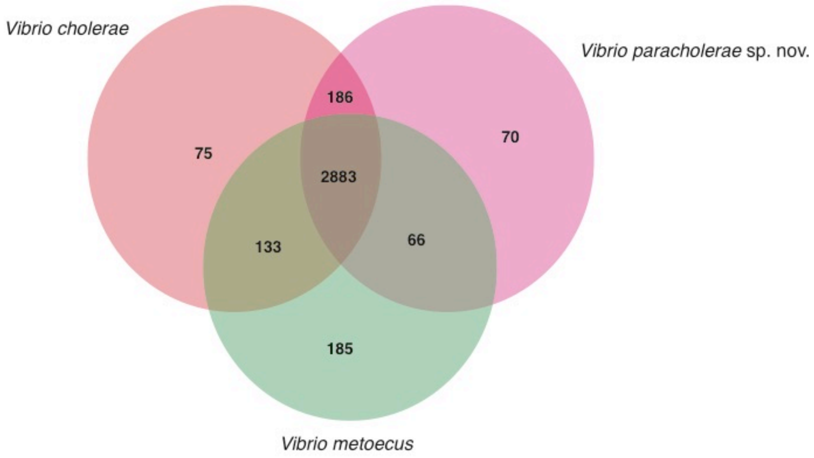

Fig. S1: Venn diagram of the orthologous protein coding gene family comparison of *Vibrio paracholerae* sp. nov. with *V. cholerae* and *V. metoecus*. Shared and unique gene clusters were calculated using OrthoVenn2 (https://orthovenn2.bioinfotoolkits.net/home).

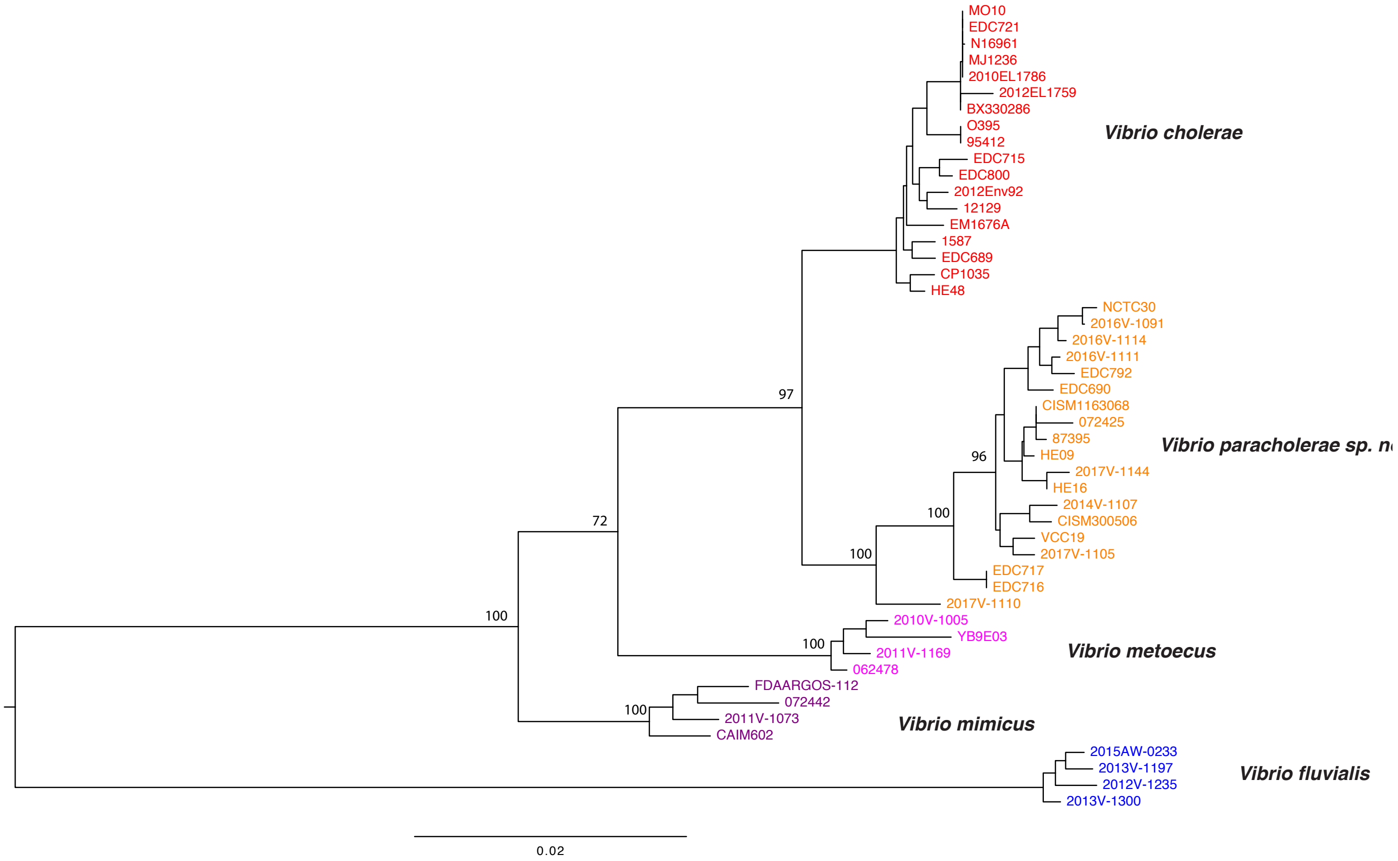

Fig. S2: Multilocus sequence analysis (MLSA) of *Vibrio paracholerae* sp. nov. and its sister species. The maximum-likelihood (PhyML) phylogenetic tree was constructed from the alignment (7392 bp) of four protein-coding housekeeping genes (*rpoA*, *rpoB*, *recA*, *pyrH*). Bootstrap support values over 70% are indicated on the corresponding nodes.

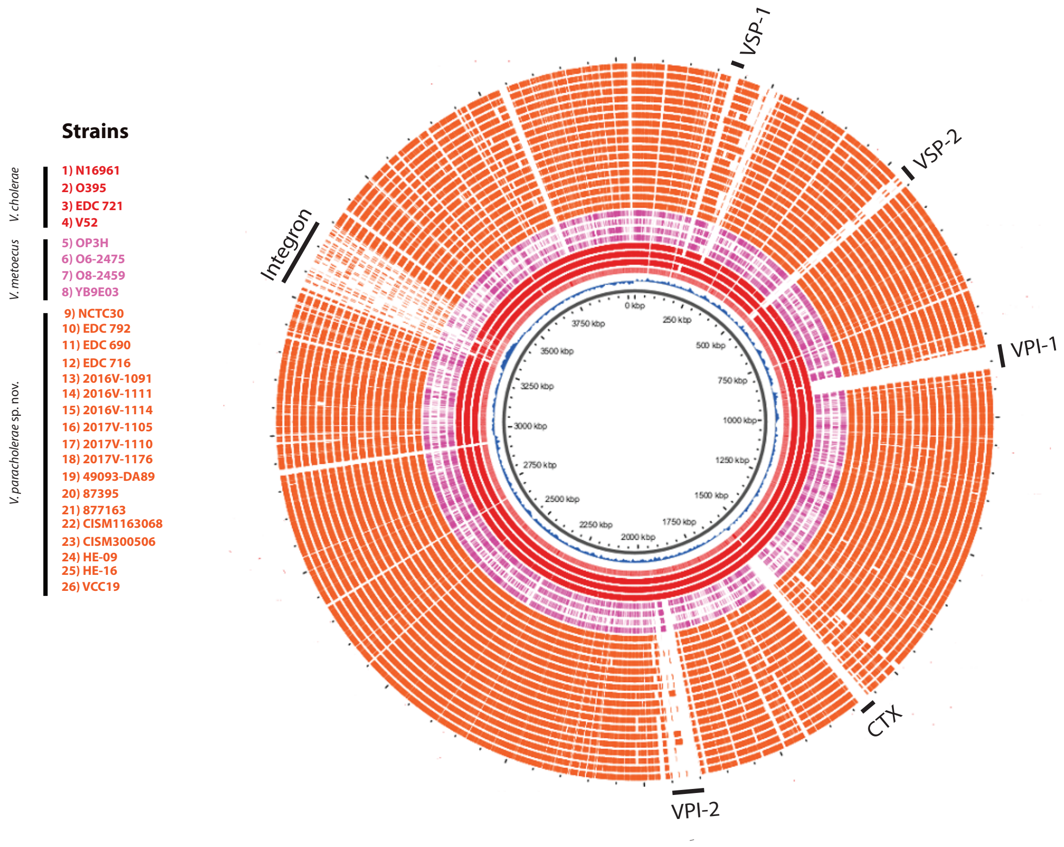

Figure S3: BLAST atlas of *V. paracholerae* sp. nov., *V. cholerae* and *V. metoecus* strains. *V. cholerae* strain N16961 was used as the reference sequence for BLASTN comparisons. Each colored ring represen a genome starting from N16961. Outermost black bars indicate the major genomic islands of *V. cholerae*. VSI Vibrio seventh pandemic island, VPI, Vibrio pthogenicity island, CTX, cholera toxin prophage.

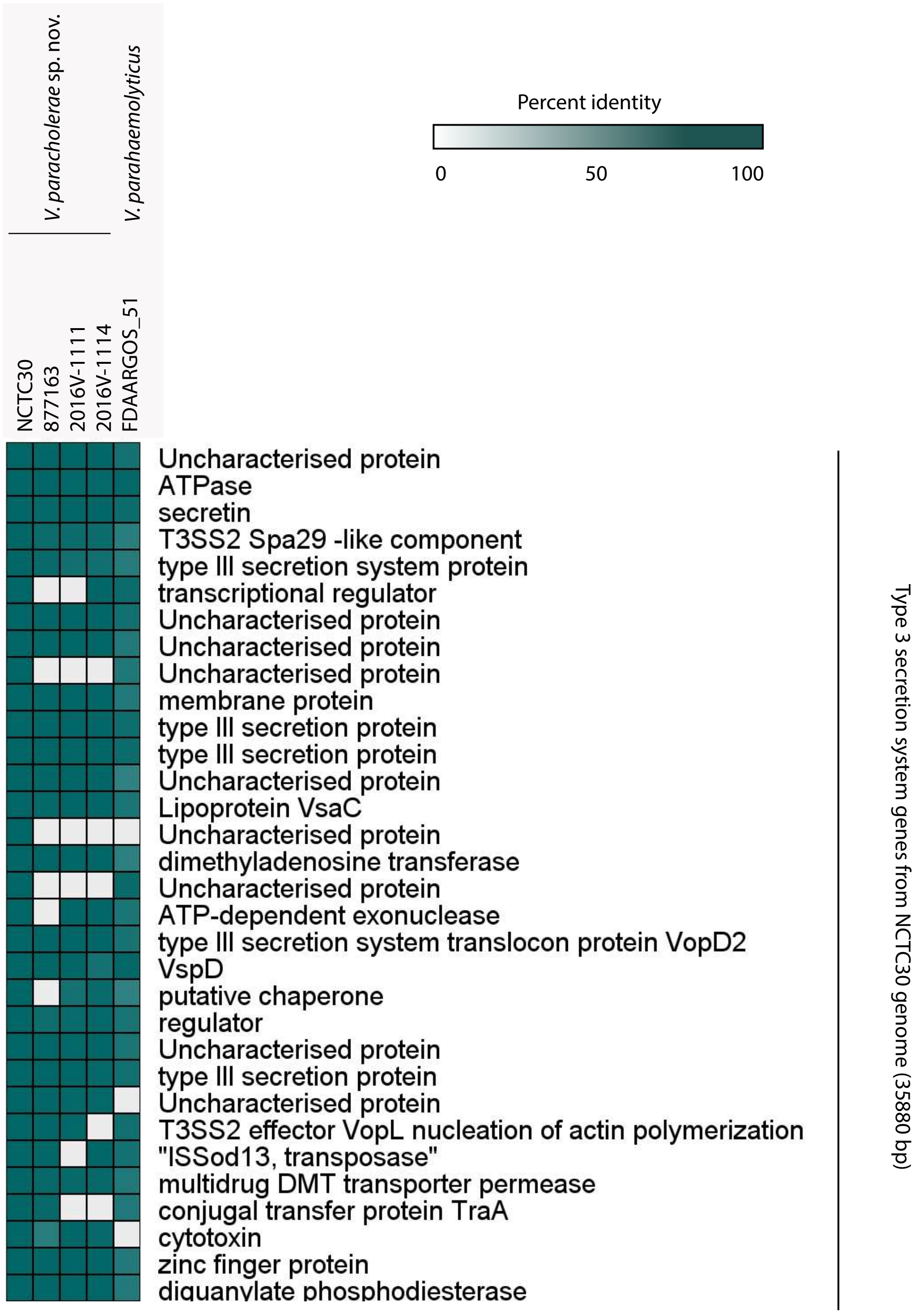

Figure S4: Comparison of T3SS island found in *Vibrio paracholerae* sp. nov. and *Vibrio parahaemolyticus*. Genes found in T3SS island of NCTC30 genome were screened in the genomes of three T3SS positive *V. paracholerae* sp. nov. strains and *V. parahaemolyticus* strain FDAARGOS_51 using BLASTN. Green box indicates presence and white box indicates absence of the genes.

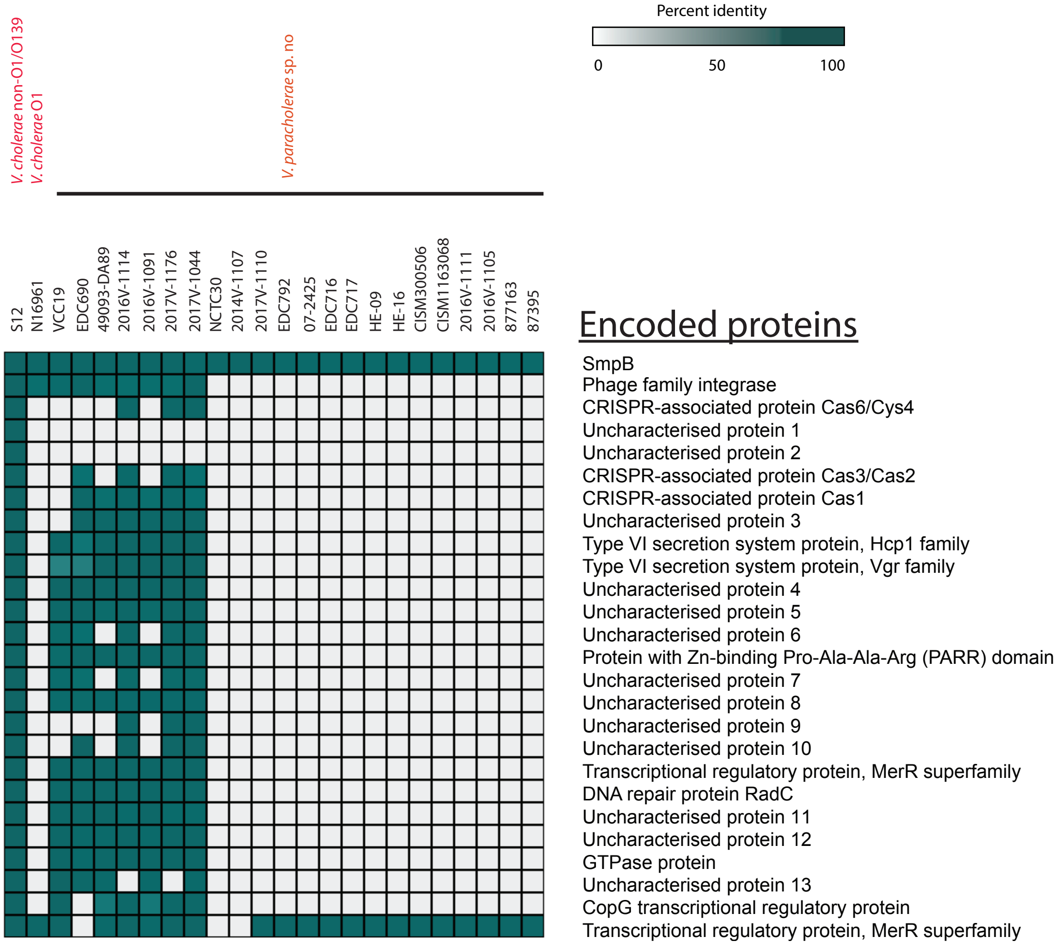

Figure S5: Presence of GIVchS12 island within VPI-1 insertion site in *V. paracholerae* sp. nov. isolates. Regions of nucleotide identity to VPI-1 and surrounding regions in *V. cholerae* N16961 are shown. Presence o Protein coding genes in GIVchS12 island described by Maurizio *et al* were looked for using BLASTN in *V. paracholerae* sp. nov. genomes. Green box indicates presence and white box indicates absence of genes.

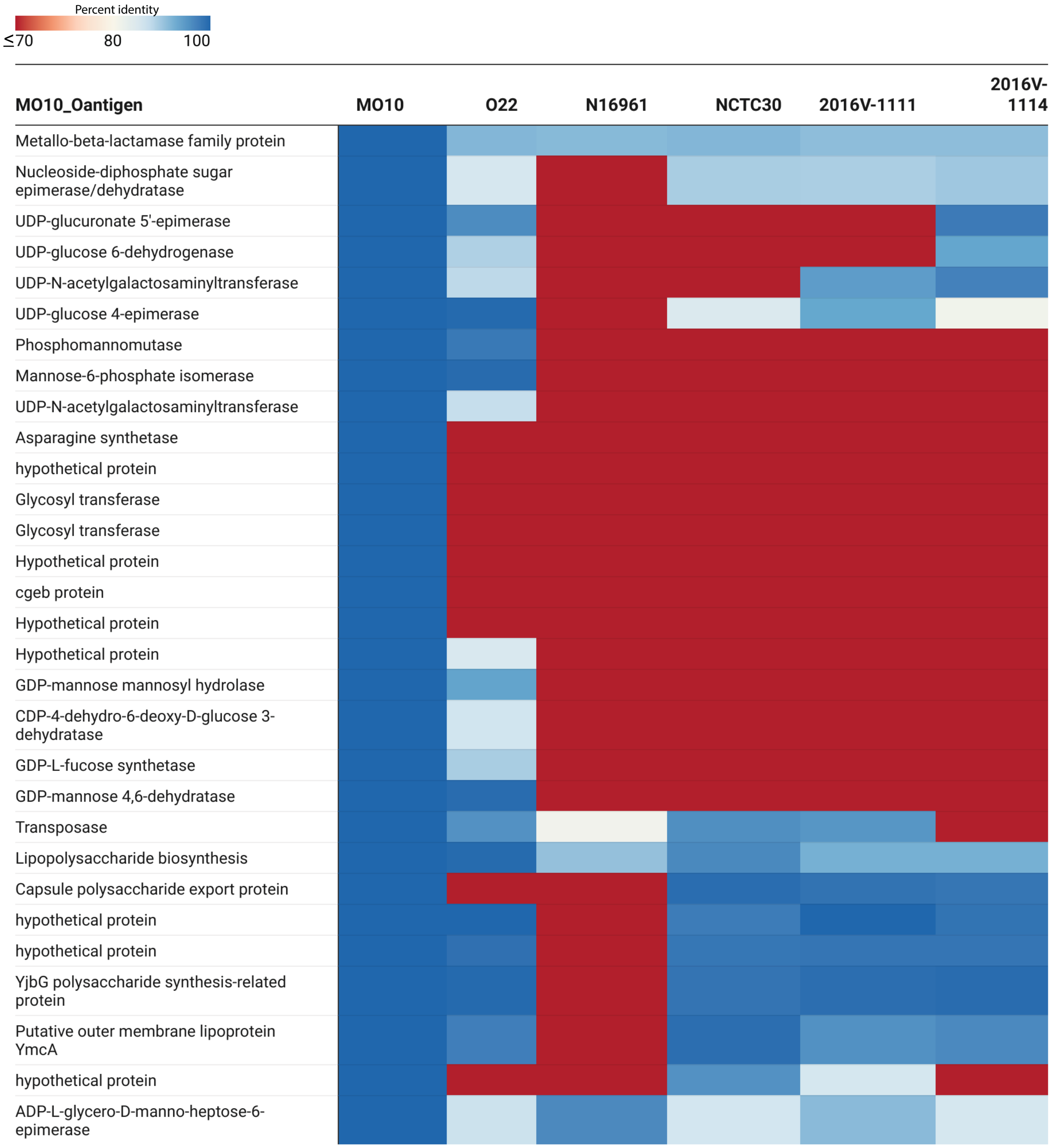

Figure S6: Comparison of O-antigen biosynthesis region of *V. cholerae* O139 with *V. cholerae* O22, *V. cholerae* O1 and *V. paracholerae* sp. nov. MO10: reference strain for *V. cholerae* O139; O22: O-antigen region for *V. cholerae* serogroup O22; N16961: reference strain for *V. cholerae* O1, NCTC30: *V.paracholerae*

sp. nov., 2016V-1111: *V.paracholerae* sp. nov; 2016V-1114: *V.paracholerae* sp. nov. O-antigen regions were extracted and compared using BLASTN.

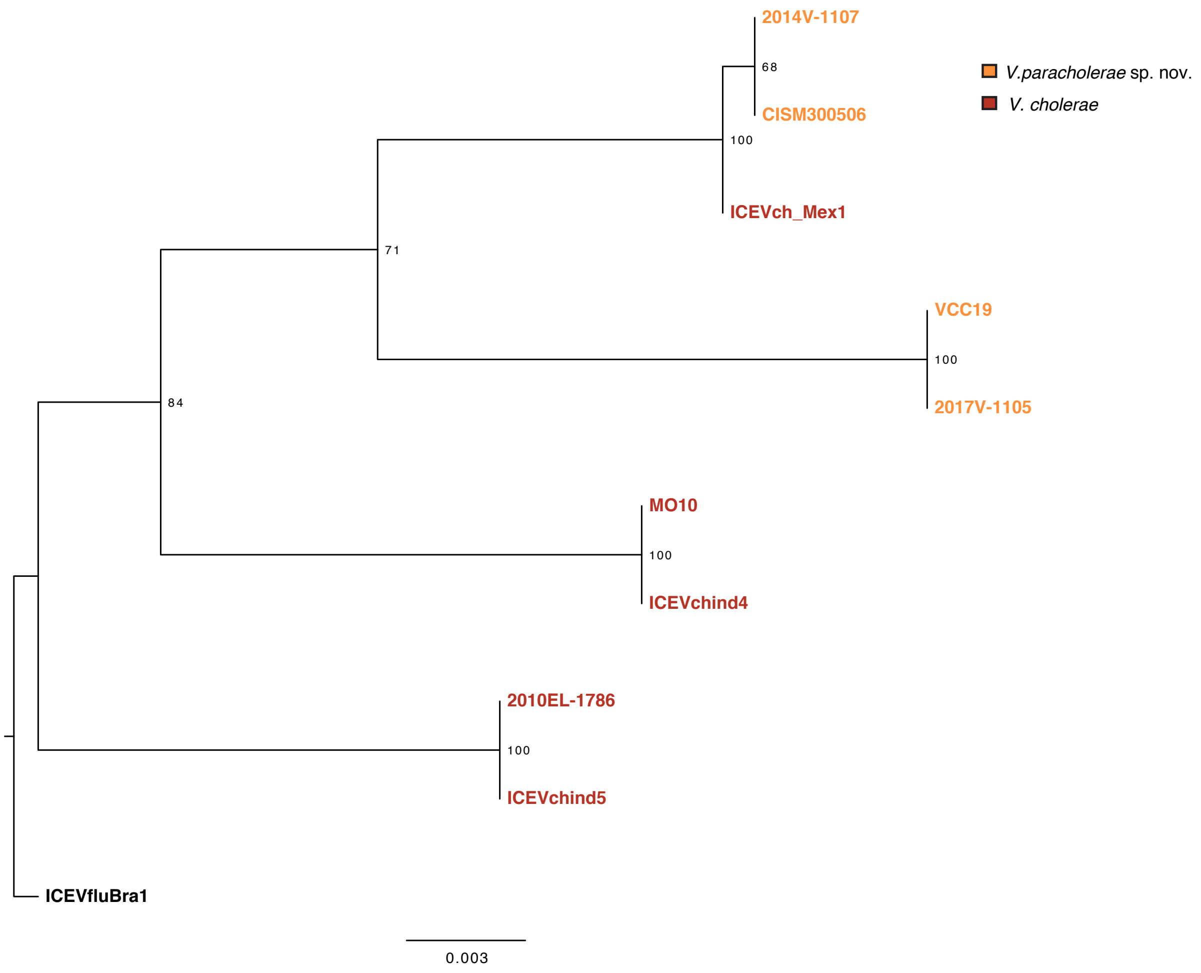

Figure S7: Maximum likelihood tree of the 1243 bp integrase gene (int) found in integrative conjugative element (ICE) of *V. paracholerae* sp. nov. and *V. cholerae* strains. Sequence of integrase gene found in *V. fluvialis* (ICEVfluBra1) was used as outgroup to root the tree. Bootstrap values are shown on the corresponding nodes.

Table S1: Physiochemical data ranges of the two sampling locations used in the study.

Location	Number of samples	Salinity range (ppt)	DO range (mg/L)	pH range	Temperature range (°C)
Dhaka	60	0-0.8	0.19-5.05	6.4-7.7	27.1-33.6
Oyster Pond	40	0-6	5.4-8.6	5.5-7.5	10.5-28

Table S2: Responses towards chemical/heavy metals of *Vibrio paracholerae* sp. nov., differentiating it from its closest relatives *Vibrio cholerae.* R: resistant; S: sensitive; ND: not done.

**Chemical/ heavy metal**	***Vibrio paracholerae* sp. nov.**				***Vibrio cholerae***			
	**EDC 690**	**EDC 716**	**EDC 792**	**2016V-1091**	**N16961**	**V52**	**YB3B05**	**YB8E08**
Cadmium chloride	R	R	R	ND	S	S	ND	ND
Sodium selenite	R	R	R	ND	S	S	ND	ND
Dichlofuanid	R	R	R	ND	S	S	ND	ND

Table S3: Presence of potential virulence traits in *V. paracholerae* sp. nov isolates in comparison to *V. cholerae* reference strains

Strain	Phylogenetic group	CTX-VPI1-VPI2	RTX toxin	SXT element	T3SS	RND efflux gene cluster	Cholix toxin (chxA)	β-lactamase
N16961	*V. cholerae* O1 El Tor (PG)	+	+	-	-	-	-	-
O395	*V. cholerae* O1 classical (PG)	+	+	-	-	-	-	-
MO10	*V. cholerae* O139 (PG)	+	+	+	-	-	-	-
12129	*V. cholerae* non-O1/O139 (non-PG)	-	+	-	+	-	-	-
VCC19	*V. paracholerae* sp. nov.	-	+	+	-	+	-	+
877163		-	+	-	+	+	+	-
EDC-792		-	+	-	-	+	+	-
EDC-690		-	+	-	-	-	+	-
EDC-716		-	+	-	-	+	-	-
EDC-717		-	+	-	-	+	-	-
HE09		-	+	-	-	+	+	-
HE16		-	+	-	-	+	-	-
CISM300506		-	+	+	-	+	+	+
CISM1163068		-	+	+	-	+	+	+
49093-DA89		-	+	-	-	+	-	+
SIO		-	+	-	-	-	+	-
2017V-1144		-	+	-	-	+	-	+

2014V-1107		-	+	+	-	+	+	-
2017V-1105		-	+	+	-	+	+	+
2016V-1114		-	+	-	+	+	-	-
2016V-1111		-	+	-	+	+	-	-
2017V-1176		-	+	-	-	+	-	+
2016V-1091		-	+	-	-	+	+	-
2017V-1110		-	+	-	-	-	-	-
87395		-	+	-	-	+	+	+
07-2425		-	-	-	-	+	-	-
NCTC 30		-	+	-	+	+	-	+

Table S4: Information of genomes falling into the *V. paracholerae* sp. nov clade disclosed in the NCI database after 2019. dDDH: digital DNA-DNA hybridization value; Clin: clinical; Env: environmental.

Strain	Origin	Source	dDDH with *V. paracholerae* sp. nov (EDC792*)*	NCBI genome accession number
N2784	China	Clin	85.2	VSHN01000030.1
N2770	China	Clin	85.7	VSHB01000078.1
N2768	China	Clin	89.4	VSGZ01000037.1
EL2338	China	Clin	89.1	VMPB01000147.1
2204	Brazil	Env	85.8	VHOF01000002.1
2290	Brazil	Env	79.1	VHOE01000001.1
N2748	China	Clin	83.7	VSGI01000001.1
EL2403	China	Clin	83.2	VMOL01000089.1
A110912Z3	Austria	Env	82.4	VIQC01000020.1
N2807	China	Clin	83	VSID01000044.1
N2795	China	Clin	83.2	VSHY01000044.1
N2794	China	Clin	83.4	VSHX01000029.1
N2791	China	Clin	83.3	VSHU01000058.1
A3_296	Brazil	Clin	80.6	QBJE01000015.1
FORC_076	South korea	Clin	86	NZ_CP026531

Table S5: Type VI secretion system effector and immunity gene combinations in *V. paracholerae* sp. nov strains. Genes in three loci are identified and named using the scheme described in Kirchberger *et al* (Kirchberger PC, Unterweger D, Provenzano D, et al. 2017. Sequential displacement of Type VI Secretion System effector genes leads to evolution of diverse immunity gene arrays in *Vibrio cholerae*. Sci Rep. https://doi.org/10.1038/srep45133).

	T6SS effector immunity locus		
Strain	aux1	aux2	main
07-2425	Aacc	Dddd	LliE*ecga
2014V-1107	Cc	Dd	Aa
2016V-1091	Ccccc	Aa	FfbE*ec
2016V-1111	Cccc	Dd	Hh
2016V-1114	Cccc	Dd	Hh
2017V-1105	Cc	Dd	Aa
2017V-1110	Cc	Aa	Gg
2017V-1144	AAcc	Dd	Ll
2017V-1176	Cccc	Aa	Aa
NCTC30	Aacc	Dd	Ii
CISM300506	Cc	Dd	Aa
CISM1163068	Aacc	Dddd	LliE*ecga
EDC 690	Aaaaac	Dd	Aa
EDC 792	Ccccc	Dd	Hh
EDC 717	Cccc	Aa	Ii
EDC 716	Cccc	Aa	Ii
49093-DA89	Aaccc	Dddd	LliE*ecga
HE-09	Aacc	Dddd	Aa
87395*	Aacc	Dddd	LliE*ecga
HE-16	Aacc	Dd	Lli
VCC19	Cc	Dd	Aa
877163	Aac	Dd	Ii

Upper case: Effector
Lower case: Immunity
* Denotes truncated E-type effector
